# BASSA: New software tool reveals hidden details in visualisation of low‐frequency animal sounds

**DOI:** 10.1002/ece3.11636

**Published:** 2024-07-03

**Authors:** Benjamin A. Jancovich, Tracey L. Rogers

**Affiliations:** ^1^ Centre for Marine Science and Innovation, School of Biological, Earth and Environmental Sciences University of New South Wales Kensington New South Wales Australia

**Keywords:** animal communication, animal vocalisation, BASSA, bioacoustics, Fourier transform, phonation, software, spectrogram, vocal production

## Abstract

The study of animal sounds in biology and ecology relies heavily upon time–frequency (TF) visualisation, most commonly using the short‐time Fourier transform (STFT) spectrogram. This method, however, has inherent bias towards either temporal or spectral details that can lead to misinterpretation of complex animal sounds. An ideal TF visualisation should accurately convey the structure of the sound in terms of both frequency and time, however, the STFT often cannot meet this requirement. We evaluate the accuracy of four TF visualisation methods (superlet transform [SLT], continuous wavelet transform [CWT] and two STFTs) using a synthetic test signal. We then apply these methods to visualise sounds of the Chagos blue whale, Asian elephant, southern cassowary, eastern whipbird, mulloway fish and the American crocodile. We show that the SLT visualises the test signal with 18.48%–28.08% less error than the other methods. A comparison between our visualisations of animal sounds and their literature descriptions indicates that the STFT's bias may have caused misinterpretations in describing pygmy blue whale songs and elephant rumbles. We suggest that use of the SLT to visualise low‐frequency animal sounds may prevent such misinterpretations. Finally, we employ the SLT to develop ‘BASSA’, an open‐source, GUI software application that offers a no‐code, user‐friendly tool for analysing short‐duration recordings of low‐frequency animal sounds for the Windows platform. The SLT visualises low‐frequency animal sounds with improved accuracy, in a user‐friendly format, minimising the risk of misinterpretation while requiring less technical expertise than the STFT. Using this method could propel advances in acoustics‐driven studies of animal communication, vocal production methods, phonation and species identification.

## Graphical abstract

The conventional method for time–frequency analysis of animal sounds can be biased and hide important details. We evaluated other methods and showed that the superlet transform outperforms the conventional method for analysis of complex, low‐frequency sounds. We also introduced BASSA, an open‐source software tool implementing this algorithm.

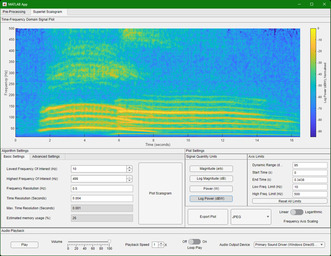

## 1 INTRODUCTION

### 1.1 Visualisation of animal sounds

Visual analysis of animal sounds is an important technique in a wide range of research areas, including sound production (Dziak et al., 2017; Fitch & Hauser, 1995; Herbst et al., 2012; Monte et al., 2020), identifying species and populations (Forti et al., 2017; Leroy et al., 2021; Wei et al., 2022), studying animal communication (Dutour et al., 2021; King et al., 2021; Vergne et al., 2009) and culture (Lachlan et al., 2018; Parker et al., 2022; Warren et al., 2020) and for estimating spatial behaviour (Blumstein et al., 2011; Mellinger et al., 2007; Rogers et al., 2013) and abundance (Dawson & Efford, 2009; Marques et al., 2013; Sugai et al., 2019; Thompson et al., 2010; Thorne & Dawson, 1974). Additionally, many bioacoustics software packages operate not on the sounds themselves, but on their visualisations (LeBien et al., 2020; Marchal et al., 2022; Mellinger & Clark, 2000; Miller et al., 2022; Popescu et al., 2013; Ulloa et al., 2021).

**FIGURE 1:**
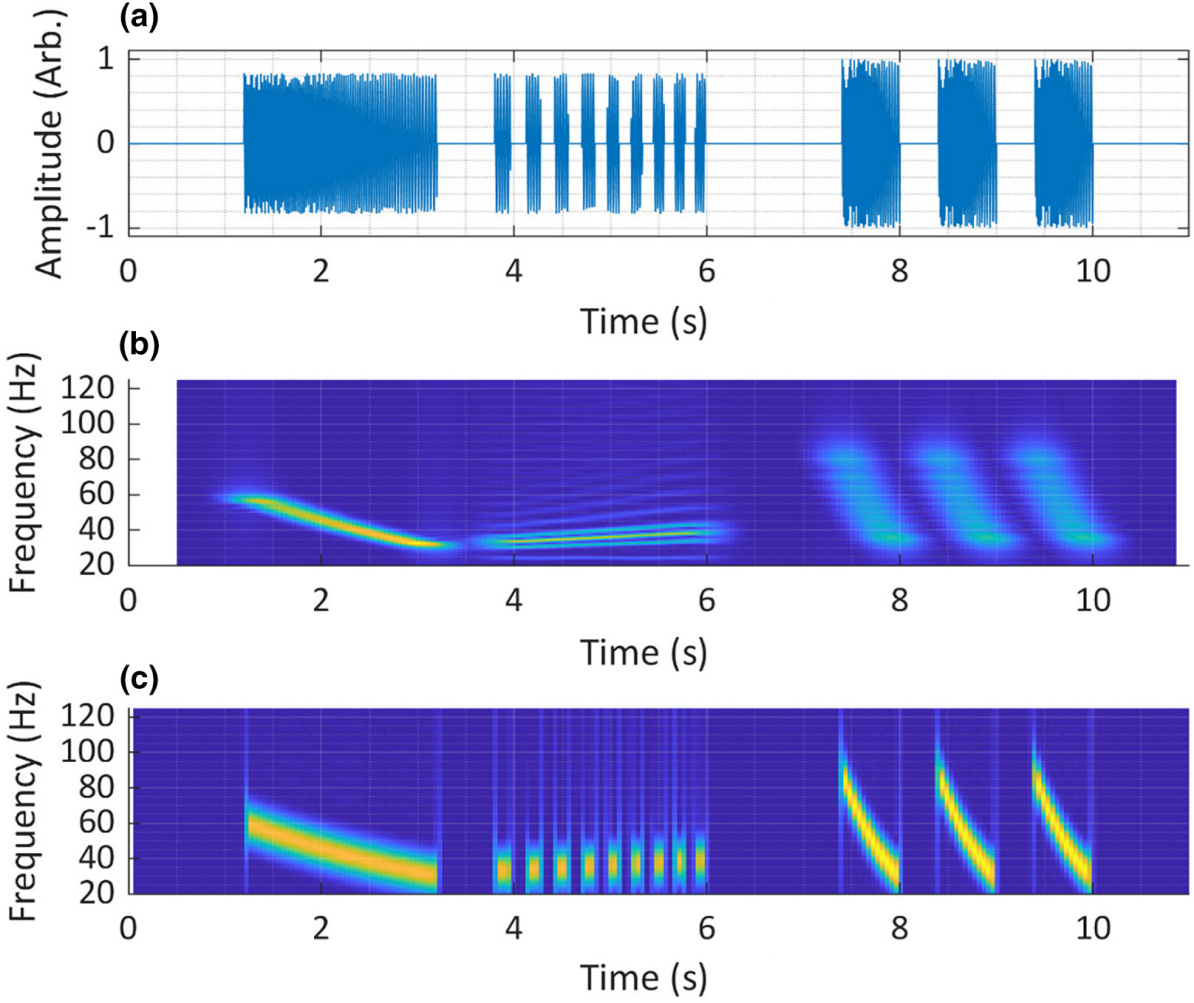
An illustration of the visualisation bias that can occur due to the trade‐off between temporal and spectral resolution. A synthetic signal with sampling frequency of 250 Hz is visualised as (a) a waveform in the time domain; (b) an STFT spectrogram biased towards frequency resolution, *n* = 250, overlap = 95% and nFFT = 2048; and (c) an STFT spectrogram biased towards time resolution, *n* = 25, overlap = 50% and nFFT = 2048. Detail is lost in both spectrograms, and neither fully captures the character of the signal.

Visualisations are also a vital tool to communicate findings and the details of animal sounds to others. An ideal visualisation should allow a reader to ‘auralise’ the animal sound, auralisation meaning in this context to form a mental impression of a sound not yet heard (Martin, 1952). An ideal visualisation therefore allows a reader to perform their own rudimentary analysis of an animal sound, without access to primary data. Short‐time Fourier transform (STFT) spectrograms are the most common type of visualisation for animal sounds, but these visualisations can obscure details and introduce bias. A deliberately exaggerated example of this bias is given in Figure 1.

### 1.2 Consequences of biased visualisation

**FIGURE 2:**
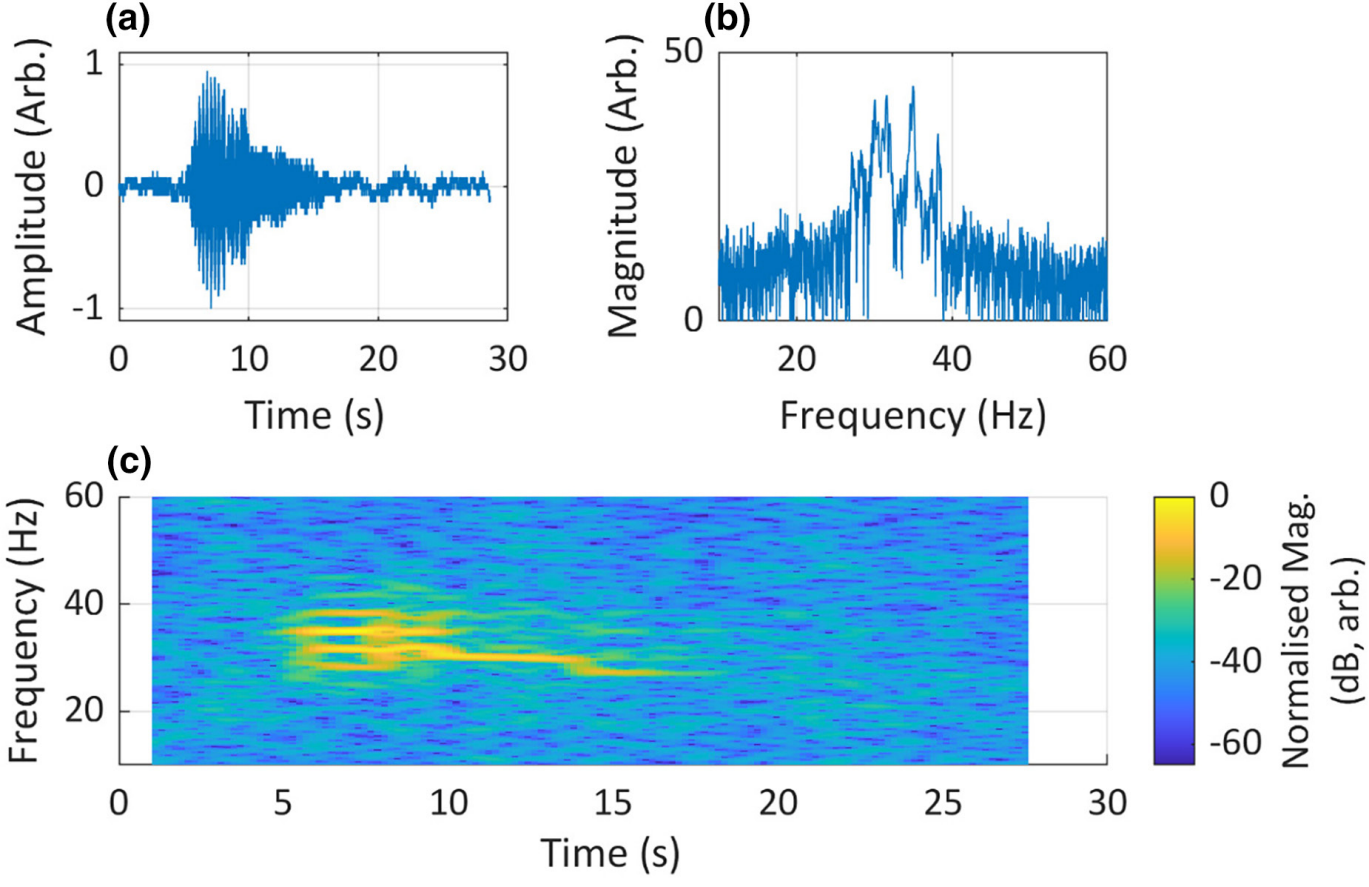
The Chagos pygmy blue whale (*Balaenoptera musculus brevicauda*) song, (a) in the time domain, (b) in the frequency domain and (c) in the time–frequency domain, visualised using an STFT short‐time spectrogram. *Fs* = 120 Hz, *n* = 500, overlap = 90%, nFFT = 4096. Recording taken at the Chagos Archipelago, Indian Ocean, isolated by Dr Emmanuelle Leroy from the CTBTO's IMS hydrophone dataset, used with permission.

STFT‐based visualisations have an inherent bias that limits their ability to convey all the information required for auralisation. This can lead to misinterpretations of acoustic structure (Herbst et al., 2013). The spectrogram's bias stems from a limited ability to accurately render both temporal and spectral details simultaneously. This is problematic for analysis of complex animal sounds, particularly when attempting to infer mechanisms of animal sound production, or when distinguishing between similar animal sounds for the purpose of identifying the source species or population. The bias in the STFT spectrogram may have been the source of a recent disagreement concerning the acoustic structure of the Chagos pygmy blue whale song (*Balaenoptera musculus brevicauda*). Blue whales produce regionally distinct, stereotyped songs (Leroy et al., 2021; McDonald et al., 2006) and a comparison of this song's unique acoustic features to other blue whale songs has indicated that the Chagos blue whales in the central Indian Ocean are a distinct acoustic population (Leroy et al., 2021). When the Chagos blue whale's song was first identified, it was proposed to be the call of an unknown whale and was termed the Diego Garcia Downsweep call (Sousa & Harris, 2015). Later, these same sounds were identified as belonging to blue whales also recorded off Sri Lanka, and the sounds were renamed as songs of the Chagos blue whale (Leroy et al., 2021). There is some disagreement, however, about the acoustic structure of the Chagos blue whale song. A study by Pinto and Chandrayadula (2021) which studied the acoustic features of the first part of this whale song (Figure 2) described it as a set of closely spaced tones, which they referred to as the comb of tones (this corresponds to unit 1 and subunits 1 & 2 in Leroy et al., 2021), with no mention of pulsing. Conversely, Sousa and Harris (2015) and, later, Leroy et al. (2021) identify this first part of the song to be amplitude‐modulated (pulsed) and not tonal. The confusion surrounding the acoustic structure of the Chagos blue whale song likely arose due to the inherent time–frequency bias of the STFT spectrogram. Sousa and Harris (2015), Leroy et al. (2021) and Pinto and Chandrayadula (2021) all provide STFT spectrograms that are biased towards retention of frequency resolution when reporting on the Chagos blue whale's song. This is not unreasonable since all three units have narrow‐band frequency components, and indeed, units 2 and 3 are entirely tonal. However, subunits 1 and 2 of the first unit of the Chagos blue whale's song are not tonal but pulsed (See Patris et al., 2019, for more information on classification of blue whale vocal units). The figures provided by Leroy et al. (2021) suggest that they were able to identify the pulsing through analysis of the time‐domain waveform (Figure 2a). Such analysis of raw waveforms in the time domain is often useful but does not appear to be common practice in bioacoustics. A visualisation biased towards frequency resolution is unable to illustrate this pulsing in the song's first unit, and so is incomplete in a temporal sense. Analysis of this unit's pulse rate and frequency spacing reveals that the additional components above and below the fundamental are, in fact, amplitude modulation sideband components. Without knowledge that this sound is pulsed, one might wrongly conclude the sound is tonal, attributing these multiple, harmonically unrelated frequency components to independent sound sources in the vocal apparatus (biphonation). The use of STFT spectrograms can therefore lead to misinterpretation and confusion.

### 1.3 The conventional visualisation method

**FIGURE 3:**
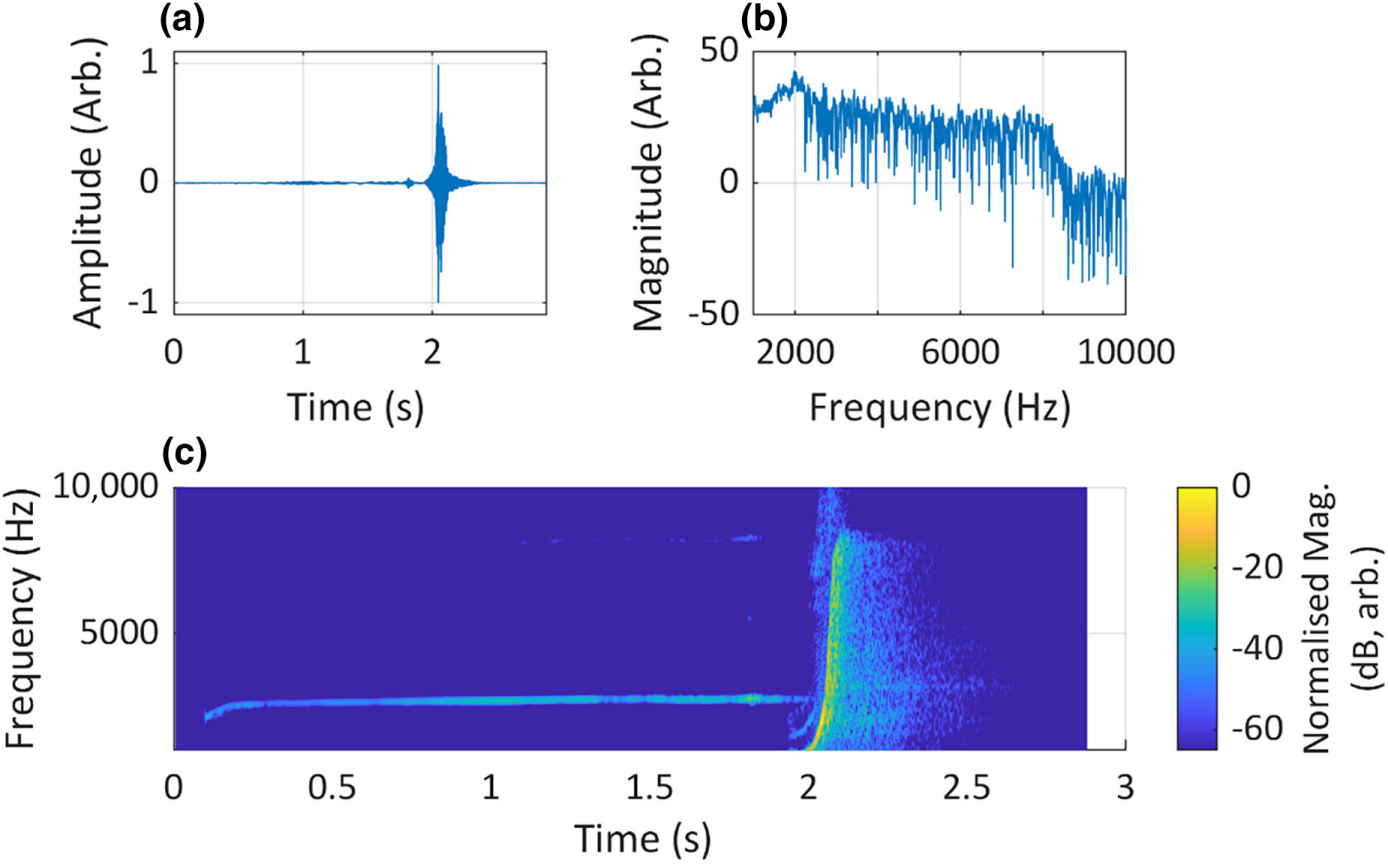
An example of a simple animal sound is the call of an eastern whipbird (*Psophodes olivaceus*). The call is visualised as (a) a waveform in the time domain, (b) a magnitude spectrum in the frequency domain and (c) a short‐time Fourier transform spectrogram in the time–frequency domain. Neither of the visualisations in (a) or (b) convey the full character of the signal. Recording is an excerpt taken from a longer recording, ‘*Psophodes olivaceus* (ML557908221)’, by David Secomb at Cardinia, Victoria, Australia, courtesy of The Macaulay Library at the Cornell Lab of Ornithology, and was used with permission.

The two most fundamental ways to visualise a sound are waveforms and magnitude spectra. Waveforms illustrate the sound in the time domain (Figure 3a) and plot changes in amplitude over time. Magnitude spectra illustrate the sound in the frequency domain (Figure 3b) and plot magnitude per frequency. For sounds consisting of simple tones that do not change over time (i.e. steady‐state signals), together these two visualisations may be adequate to convey the acoustic features of the sound. Animal sounds, however, are rarely if ever, steady state. They often feature complex spectral and temporal structures, with multiple overlapping tones fluctuating in frequency and amplitude over time (Baotic et al., 2014; Cazau et al., 2016; Gonzalo‐Tarodo et al., 2020; Hedwig et al., 2021; Leroy et al., 2021; Zhang et al., 2017). Effective visualisations of animal sounds, therefore, must illustrate the amount of acoustic energy, where that energy occurs in the frequency spectrum and when it occurs in time. This type of visualisation is known as ‘time‐frequency’ (TF) analysis. The most common form of TF analysis is the spectrogram, developed by Potter (1945), and the algorithm underlying the modern implementation of the spectrogram is the short‐time Fourier transform (STFT) (Zölzer & Amatriain, 2002, pp. 15–17). The STFT operates by segmenting a time‐domain signal into consecutive ‘frames’ of a short duration, relative to the entire signal. The Fourier transform of each frame is computed to produce a magnitude spectrum, and these spectra are plotted as vertical slices of a heatmap, with their Y‐axis values (the signal intensity) illustrated using colours (Figure 3c), and expressed as magnitude or power, either linear or log‐scaled (dB). In bioacoustics, time is most often plotted along the X‐axis, and frequency along the Y‐axis.

### 1.4 Time–frequency bias in the STFT

The time–frequency bias in the STFT spectrogram is a result of the Gabor–Heisenberg uncertainty principle. This states that a signal's precise location in both frequency and time cannot be computed simultaneously (Gabor, 1946). The consequence is that the results of an STFT vary dramatically depending on how the algorithm has been configured, and particularly on the length of the frames. Longer frames result in bias towards better frequency resolution, but poorer time resolution, and shorter frames result in the inverse (Potter, 1945). The shape of the ‘window function’ used to smooth each frame also introduces errors to the result. A wider window function (when plotted in the time domain), will bias the result towards better frequency resolution, while a narrow window will bias towards time resolution. The shape of the window function when transformed to the frequency domain also has consequences, with the width of the main lobe influencing frequency resolution. When compared with a wide main lobe, a narrow main lobe will have better frequency resolution but increased spectral blurring or ‘leakage’ between adjacent frequencies. Spectral leakage also occurs with increases in the magnitude of any side lobes. Finally, the amount of time overlap between successive frames has an effect, with increased overlap resulting in improved temporal resolution and decreased spectral leakage. The parameters used to compute an STFT can therefore have a profound influence on the appearance of, and ultimately, the conclusions that can be drawn from the resulting visualisation.

The STFT was first used to study animal sounds in 1971 (Coutlee, 1971), and has since become a ubiquitous feature of research in bioacoustics (Baotic et al., 2014; Boucher et al., 2020; Bussmann et al., 2021; Cazau et al., 2016; Davenport et al., 2022; Dutour et al., 2021; Gonzalo‐Tarodo et al., 2020; Jégh‐Czinege et al., 2020; Leroy et al., 2021; Malige et al., 2020; Mann et al., 2021; McDonald et al., 2009; Odom et al., 2021; Stoeger et al., 2014; Teixeira et al., 2022; Thode et al., 2017); however, the TF bias is rarely discussed in the bioacoustics literature. TF analysis is used widely in engineering, where it is generally acknowledged that this bias imposes a limit on the STFT spectrogram's accuracy when applied to complex signals, especially at low frequencies. Previous research has described the STFT spectrogram's limitations in seismology (Leśniak & Niitsuma, 1996), electro‐mechanical fault detection and diagnosis (Dong & Chen, 2012; Feng et al., 2013; Panagiotou et al., 2019), electroencephalography (Arts & van den Broek, 2022) and magnetic resonance and computed tomography imaging (Zhu et al., 2003). Many of these fields have adopted other methods. While there has been at least one study highlighting the issues with the STFT spectrogram in the study of animal sounds (Brumm et al., 2017), this remains the most common method in the biological sciences (Baotic et al., 2014; Benko & Perc, 2009; Bonnefond et al., 2020; Bussmann et al., 2021; Cazau et al., 2016; Davenport et al., 2022; Dutour et al., 2021; Forti et al., 2017; Gonzalo‐Tarodo et al., 2020; Jégh‐Czinege et al., 2020; Leroy et al., 2021; Malige et al., 2020; Mann et al., 2021; Maruska & Mensinger, 2009; McDonald et al., 2009; Nelson et al., 2011; Odom et al., 2021; Rice & Bass, 2009; Salas et al., 2018; Staniewicz et al., 2023; Stoeger et al., 2014; Sueur et al., 2011; Teixeira et al., 2022; Thode et al., 2017).

Given the inherent bias in the STFT, and the substantial influence algorithm parameters can have on the resulting visualisation, the selection of these parameters should be undertaken with care. Parameter optimisation is dependent on the expertise and diligence of the researcher and requires some prior knowledge of acoustic features of the animal sound. The choice of parameters is also influenced by the aims of the analysis. For example, research goals may dictate that high‐frequency resolution is of primary importance, and this would necessitate discarding temporal detail from visualisations during initial analysis and publication. It is, therefore, possible that STFT visualisations may routinely be misinterpreted both by researchers and readers.

An optimal time–frequency analysis method for animal sounds could be defined as one that is able to produce visualisations with minimal time–frequency bias that requires no specialist expertise to interpret, and whose analysis parameters require minimal specialist expertise to configure. Such optimal methods would benefit researchers through faster, easier and more accurate analysis of animal sounds, and would better equip readers to interpret and evaluate the conclusions of published work and to draw inspiration for future work. Further advances in methods for time–frequency analysis therefore have real benefits for biological and ecological research.

### 1.5 Advances in time–frequency analysis

Several advances have been made in time–frequency analysis since the 1940s, and these include a variety of STFT‐based methods, as well as wavelet‐based methods such as the continuous wavelet transform (CWT). The Wigner–Ville distribution function (Mallat, 1999, p. 4) uses auto‐correlation and the Fourier transform to perform TF analysis with high resolution in both time and frequency. Its main limitation is strong cross‐term contamination, which can corrupt results for complex signals. Another newer method is the Choi–Williams distribution function (CWDF) (Choi & Williams, 1989), which attempts to suppress cross‐terms. Unfortunately, this results in the suppression of real signal components (Papandreou & Boudreaux‐Bartels, 1993), risking the loss of information of interest from the result. The multitaper spectrum (Xiao & Flandrin, 2007) is another TF analysis method, which in the simplest case, involves taking the mean of multiple STFTs made with a variety of window functions, referred to as tapers. This method does reduce spectral curvature bias and spectral leakage but features increased complexity and requires substantially greater expertise to use effectively. Additionally, the averaging used to suppress random variability in this method can introduce bias (Prieto et al., 2007).

An additional method that is of interest is empirical mode decomposition (EMD) (Huang et al., 1998). This method differs in that it does not involve moving to the time–frequency domain, but rather decomposes the recording into a plurality of time‐domain waveforms called intrinsic mode functions (IMFs). IMFs are sorted in order of their contribution to the recording's total energy and can be thought of as time‐domain representations of each of the signal's frequency components. EMD is a useful time–frequency analysis method, but since its results are visualised as a series of waveforms, interpretation may be less intuitive than for a spectrogram. Additionally, it can be difficult to discern which IMFs correspond to signal and which correspond to background noise. For a more complete discussion of the most common time–frequency analysis methods, please see the Appendix S1.

Common among newer methods is increased complexity, both computationally, and in terms of the expertise required to use them effectively, when compared with the STFT. This is likely one of the reasons that the STFT remains the dominant method for visualising animal sounds. The STFT, however, introduces TF bias, and while relatively user‐friendly, it does still require a non‐trivial degree of expertise to produce reliable results. There is, therefore, a real need for a method that offers robustness, simplicity, familiarity, interpretability, accuracy and ease of use for researchers without specialist signal processing expertise. Due to the broad use of TF visualisations in acoustic ecology and bioacoustics, this gap has consequences for the study of animal communication, behaviour, culture, evolution, vocal production anatomy and physiology, density estimation, population distribution and taxonomy.

The superlet transform (SLT) is an optimised generalisation of the CWT introduced in 2021 for the analysis of brainwave data (Moca et al., 2021). The SLT operates by taking the geometric mean of a plurality of CWTs, each having a differing number of cycles in its mother wavelet (Moca et al., 2021). The CWT's time and frequency resolution are determined primarily by the number of wavelet cycles, so the SLT can be described as the average of many wavelet transforms, each having different time–frequency resolution. The intended outcome is improved and constant time–frequency resolution across the entire analysis bandwidth. Compared to the STFT, the SLT does have increased computational cost, and this will affect the time to compute TF visualisations, however, since compute time is implementation dependant, it is not evaluated in the present study. Following its original application in neuroscience (Moca et al., 2021), the SLT has been evaluated in several other applications, including the automated diagnosis of Parkinson's disease using human speech signals (Bhatt et al., 2023), automated diagnosis of cardiac arrhythmias (Tripathi et al., 2022), remote hydrocarbon reservoir detection in seismic surveying (Tian et al., 2022) and for mechanical fault diagnosis of rolling bearings in wind turbines (Yi et al., 2023). All these studies found the SLT to outperform other TF analysis methods.

The STFT underpins the time–frequency visualisation of animal sounds in many fields of bioacoustics. Here, we investigate whether other analysis methods can produce visualisations of low‐frequency animal sounds with improved accuracy. First, we examine the performance of four different TF analysis methods (SLT, CWT and two STFTs) in visualising a synthetic sound with known acoustic characteristics and measure the error in each visualisation. Second, we develop and provide an open‐source GUI‐based software application that performs TF analysis and generates customisable visualisations using the best‐performing method, without the need for the user to write computer code. Third, we identify whether the best‐performing method provides TF visualisations that accurately render the acoustic features of complex, low‐frequency animal sounds.

## 2 METHODS

### 2.1 Quantitative evaluation

#### 2.1.1 Experimental design

**FIGURE 4:**
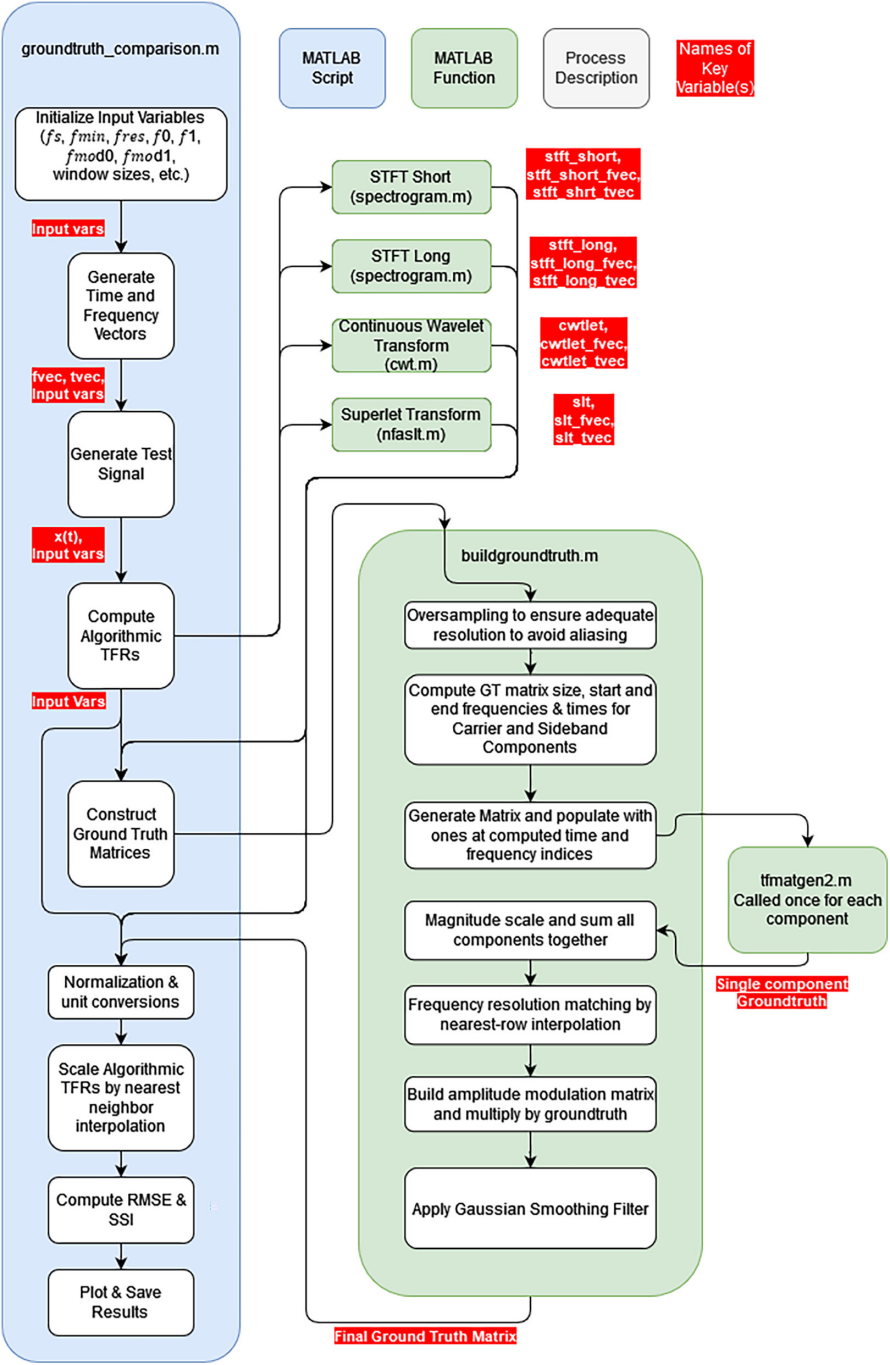
Programmatic flow diagram of the MATLAB code written to perform quantitative analysis and comparison of TFR methods.

The short‐time Fourier transforms (STFT), continuous wavelet transform (CWT) and superlet transform (SLT) all generate time–frequency representations (TFRs) as two‐dimensional matrices of signal intensity values. The row indices of these matrices correspond to frequency, and column indices to time. Each algorithm was tested using a synthesised signal resembling a pulsed, low‐frequency animal sound. Using a synthetic test signal with known time–frequency properties allowed us to define a synthetic ‘ground truth’ TFR, serving as a benchmark for comparison with the TFRs generated by the four methods (the algorithmic TFRs). We evaluated the agreement of each algorithmic TFR with the ground truth using statistical measures of image similarity and error. The intensity values in these TFRs were linearly scaled, unitless magnitude and normalised to a maximum value of 1. Custom MATLAB code was used to generate the test signal, construct the ground truth TFR, implement the TF analysis algorithms and measure each algorithm's performance (Figure 4). While this summary covers key details, some have been omitted for brevity; these are provided in the Appendix S1. The code is available in its entirety at the project's GitHub Repository.

Figure 4 shows a programmatic flow diagram that illustrates the MATLAB procedure that ran the quantitative evaluations.

#### 2.1.2 Algorithm parameters

Two instances of the short‐time Fourier transform (STFT) were compared, one configured to prioritise time resolution (‘short‐STFT’) and the other to prioritise frequency resolution (‘long‐STFT’). The continuous wavelet transform (CWT) and superlet transform (SLT) were tuned for the best possible performance in both domains. The sampling frequency, *fs* = 250 Hz, low‐frequency analysis limit *fmin* = 10 and high‐frequency analysis limit *fmax* = *fs*/2 were all held constant between the four analysis algorithms. The fast Fourier transform length nFFT for both STFTs was defined as follows:

(1) $$\text{nFFT}=2\left(\frac{\mathrm{fs}}{2}/f_{\mathrm{res}}\right)$$

where *f*_res_ is the target frequency resolution, 0.2 Hz. The long‐STFT used a Hamming window, *fs* = 250, *n* = 250 and overlap = 75%. Short‐STFT used a Hamming window, *fs* = 250, *n* = 50 and overlap = 75%.

The CWT method used a symmetric Morse wavelet with a time‐bandwidth product of 120, and the CWT filter bank had 48 voices per octave.

The SLT was configured for an initial superlet of three cycles, a superresolution order interval of 10:40 and was run in multiplicative mode. Frequency resolution was *f*_res_ = 0.2 Hz.

#### 2.1.3 Test signals, ground truth and performance measures

**FIGURE 5:**
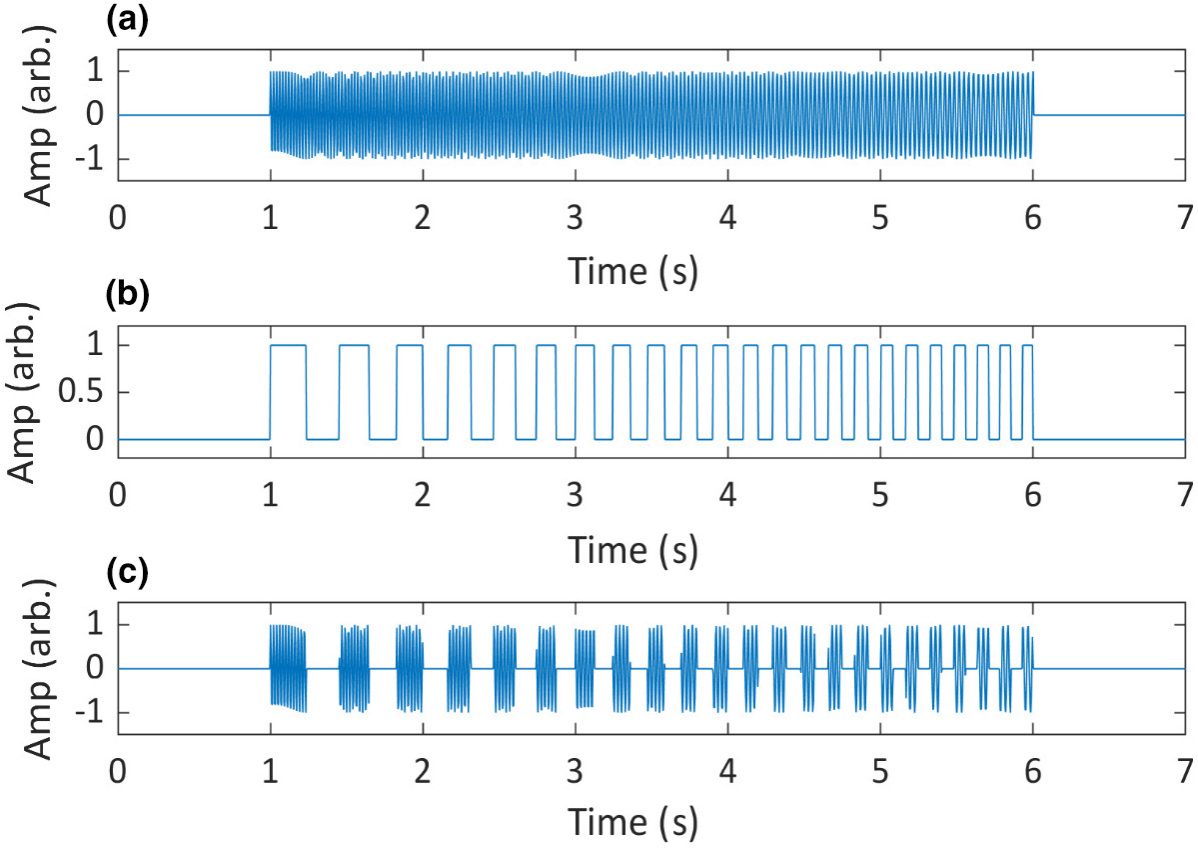
Construction of the synthetic test signal represented in the time domain. Panel (a) shows the carrier signal x_𝑐_ (𝑡), panel (b) shows the amplitude modulation signal 𝑥_𝑚_ (𝑡) and the bottom panel, (c) shows the final test signal 𝑥 (𝑡).

The time‐domain test signal was a frequency‐ and amplitude‐modulated (AM) sine wave (Figure 5a). The carrier signal was frequency modulated linearly from 30 to 50 Hz. The AM signal was a square wave, with linear frequency modulation from 2 to 7 Hz. A square wave was chosen for the AM signal, as this would produce more sideband components than a sinusoidal modulation, therefore rendering a more complex test signal. It should be noted that this represents a more extreme case of AM than is usually seen in animal sounds (Herbst et al., 2012).

**FIGURE 6:**
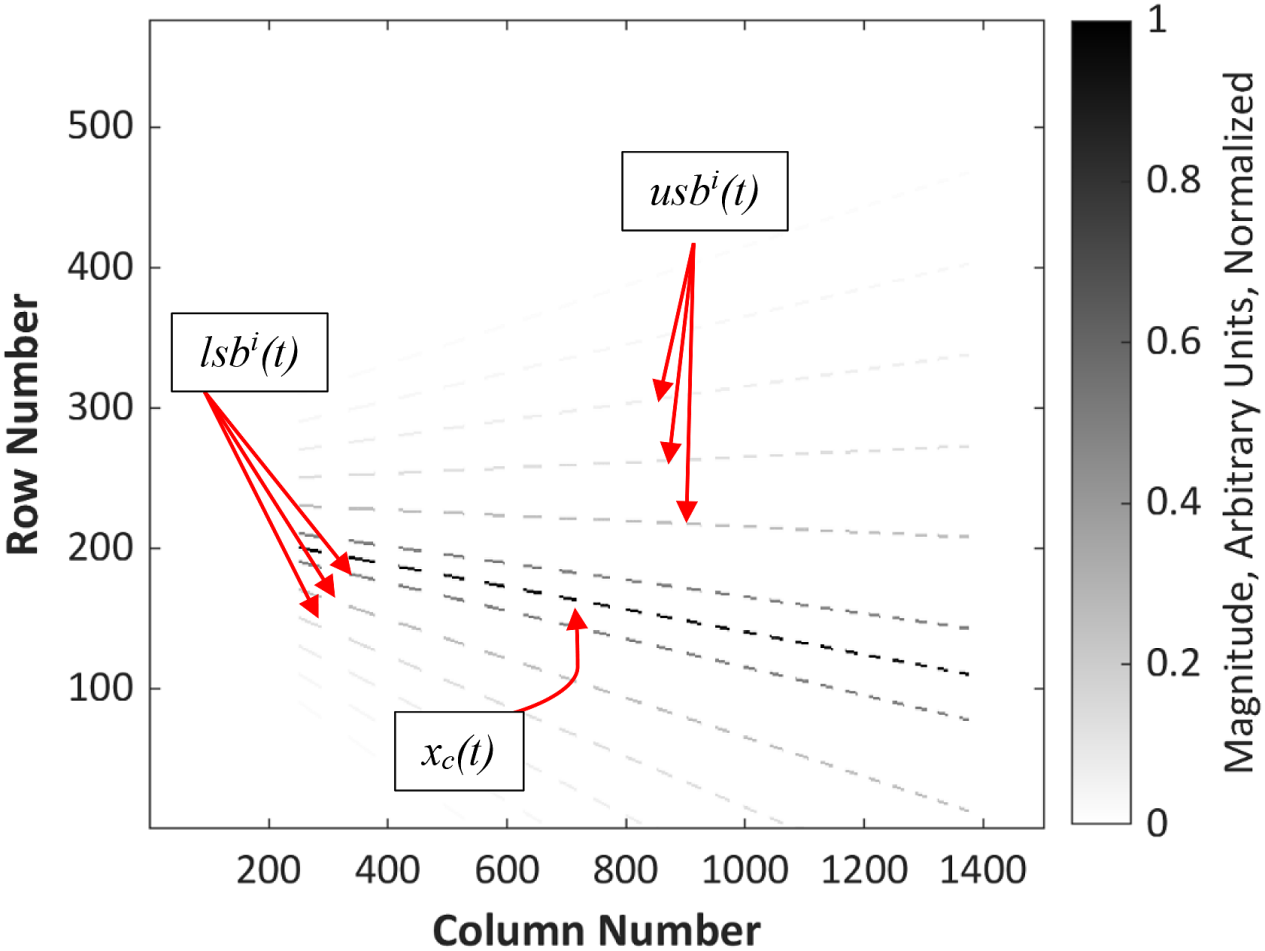
An example ground truth matrix for the carrier signal 𝑥_𝑐_ (𝑡), and the upper and lower sideband components 𝑢𝑠𝑏^𝑖^ (𝑡) and 𝑙𝑠𝑏^𝑖^ (𝑡) for 𝑖 = [1:3]. *Fs* = 250 Hz, *fmin* = 10 Hz, *fmax* = *fs*/2, *f*_0_ = 50, *f*_1_ = 30, *fmod*_0_ = 2, *fmod*_1_ = 7 and *f*_res_ = 0.2 Hz.

A ‘ground truth’ time–frequency representation was generated to represent this test signal in the time–frequency domain (Figure 6). This was designed to be a near‐perfect visualisation against which the methods under evaluation would be compared. The ground truth TFR visualised the test signal as an amplitude‐modulated (AM) carrier tone, with smooth, downward frequency modulation (FM), accompanied by 10 discrete AM sideband components, also smoothly frequency modulated. Outside the 11 tonal components, all regions of this TFR, including the spaces in between AM pulses, were visualised as silence.

The time–frequency representation (TFR) returned by each method was scored on its agreement with the ground truth matrix. Two performance measures were used; root mean square error (RMSE) and structural similarity index (SSI).

Three different formulations of root mean square error were calculated. First, the RMSE was calculated between the elements of each row of the algorithmic TFRs and ground truth TFR, which represented the error for each sampled frequency, then the ‘Mean Spectral RMSE’ was calculated by taking the mean of RMSE for all rows. The same was repeated for each column to derive the ‘Mean Temporal RMSE’. Third, a ‘Total RMSE’ was calculated, which was the root mean square error between all elements of the algorithmic TFRs and the elements of their corresponding ground truth TFRs.

Structural similarity index (SSI) is a statistical measure of similarity commonly used in image processing and computer vision fields. Structural similarity index was calculated by treating the TFR matrices as greyscale images, and following Wang et al. (2004), calculated the similarity between each algorithmic TFR and its corresponding ground truth.

Full details regarding the synthesis of the test signal, construction of the ground truth and the formulation of performance measures are given in the Appendix S1. A MATLAB script was used to generate the test signal and ground truth TFR and to perform the evaluations. This script is available in the project's GitHub Repository.

To compare how the acoustic structures of complex animal sounds are rendered by the four analysis methods, we examined TF visualisations of the song of the Chagos blue whale (*Balaenoptera musculus brevicauda*), the rumble of the Asian elephant (*Elephas maximus*), the song of the eastern whipbird (*Psophodes olivaceus*), the grunts of the southern cassowary (*Casuarius casuarius*), the grunts of the mulloway fish (*Argyrosomus japonicus*) and the mating growl of the American crocodile (*Crocodylus acutus*). The animal sounds were analysed using a custom MATLAB program available at the project's GitHub Repository. Original recording details, sources, authors and locations (where available) and the TF visualisation settings are listed in figure captions in Section 3.3.

The whale, elephant, cassowary, mulloway and crocodile were chosen as test cases as their vocalisations are both low frequency and complex in their temporal and spectral structures, making them ideal candidates for analysis with the SLT. The whipbird was included as a challenging test case, as it covers a very large bandwidth, is a high‐frequency sound and is not overly complex in structure. This was included to illustrate results for sounds that are not ideally suited for SLT analysis. No anurans or insects were included as their sounds tend to contain energy mainly above 1 kHz, and this is not where the SLT is best utilised.

### 2.2 Software application

Bio‐Acoustics Superlet Scalogram Analyser (BASSA), is an open‐source, desktop software application that implements the superlet transform (SLT), providing a simple, GUI‐based tool to generate high‐fidelity visualisations of low‐frequency animal sounds with complex temporal and spectral structures. To our knowledge, the only publicly available implementations of the SLT require the user to write at least some computer code. BASSA was designed to improve accessibility of the SLT for researchers who do not possess the necessary coding expertise to use those existing implementations. Additionally, the tool was designed with zero software dependencies, eliminating the need for expensive commercial software licences (as required for the existing MATLAB implementation of the SLT) or complicated software development environment setup (as is necessary for the existing Python implementation). The BASSA application is open source, however, MATLAB is not, so while a MATLAB licence is not needed to use BASSA as an end user, persons wishing to make contributions to the BASSA codebase will need a licence for MATLAB. Currently, BASSA is limited to the Windows platform.

Due to the computational complexity of the SLT, BASSA has limitations on the maximum sample rate (1 kHz) and duration of sounds (80 k samples) it can analyse. Due to these limitations, the current implementation of BASSA is aimed towards the analysis of low‐frequency animal sounds such as those of cetaceans, fish, large terrestrial mammals and reptiles and some avian species. The target audience for this software is researchers who do not write code, but who prefer a simple, GUI‐based tool for high‐resolution time–frequency analysis, to inspect fine‐grained details of a single instance of an animal sound. We envisage BASSA being most useful in areas of study such as phonation, vocal production methods and species identification. The software was not designed with ecoacoustics or passive acoustic monitoring in mind, as these applications deal with significantly longer duration recordings than BASSA can presently accommodate. It is, however, worth noting that these limitations are not inherent in the SLT itself, but rather are features of its current implementation in BASSA, and should not detract from the broader utility of the SLT method.

**FIGURE 7:**
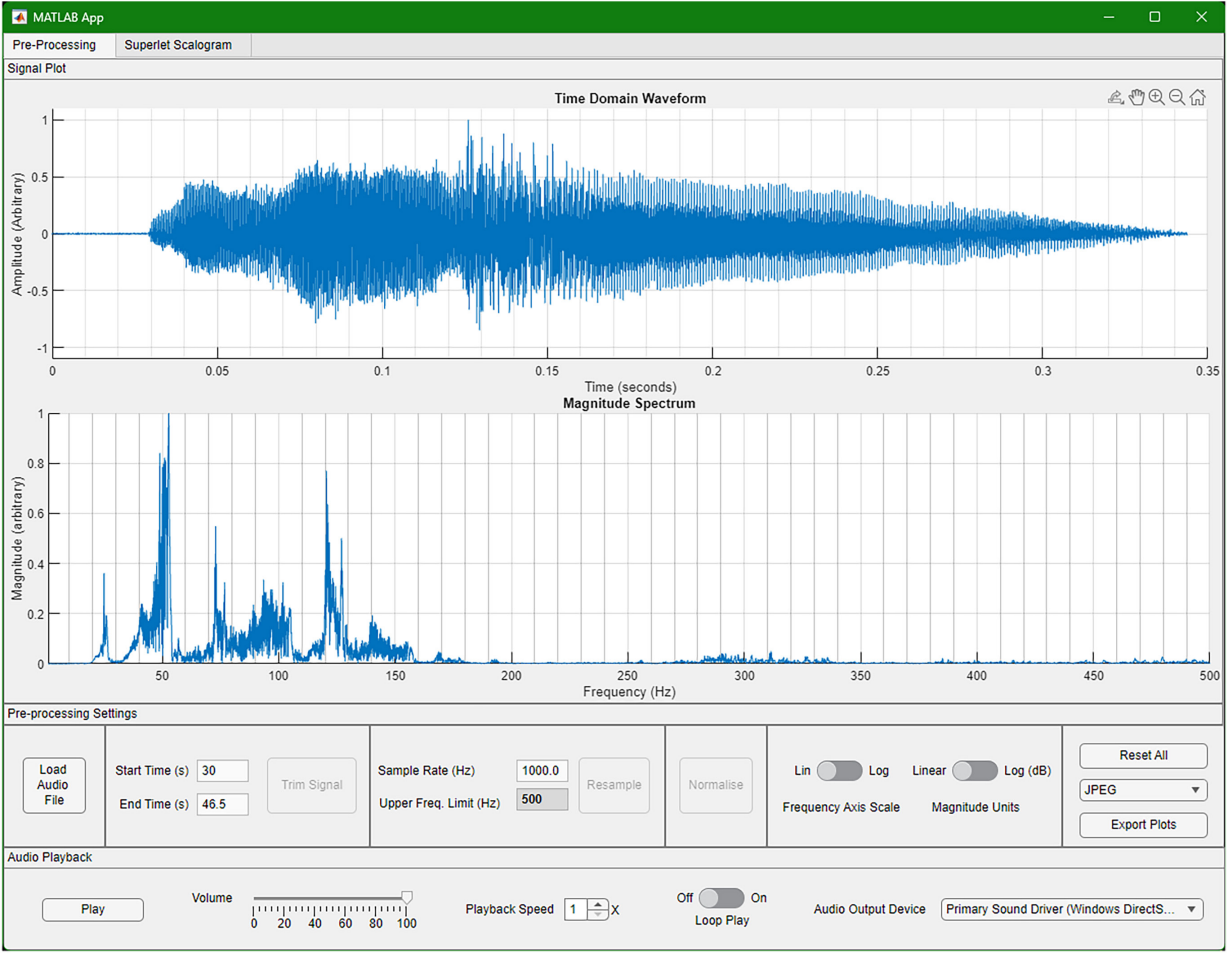
The BASSA pre‐processing screen. This screen allows for trimming, resampling, amplitude normalisation and playback, as well as time‐domain and frequency‐domain visualisation export.

**FIGURE 8:**
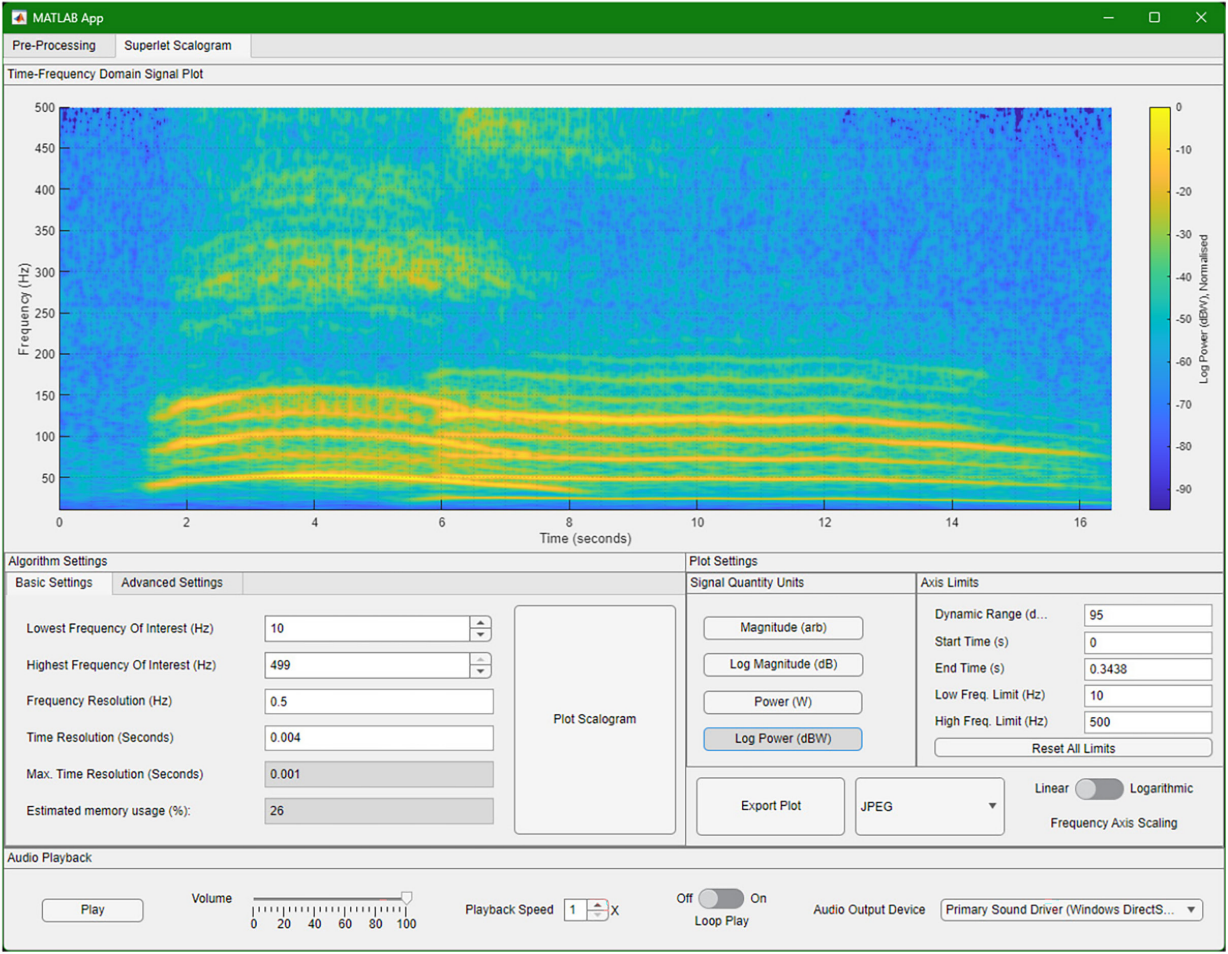
The BASSA superlet scalogram screen. This screen allows for configuration of the SLT, figure generation as well as post‐processing and export of the visualisation.

For a complete description of BASSA's capabilities and limitations, and the complete user guide, see the Appendix S1. Figures 7 and 8 show the two main screens of the BASSA graphic user interface. Compiled installers and source code for BASSA are available at the BASSA GitHub Repository. For those interested in implementing the SLT for themselves, the source code, provided by the original developers (Moca et al., 2021), is available here: https://github.com/TransylvanianInstituteOfNeuroscience/Superlets.

## 3 RESULTS

### 3.1 Quantitative evaluation – synthetic test signal

**FIGURE 9:**
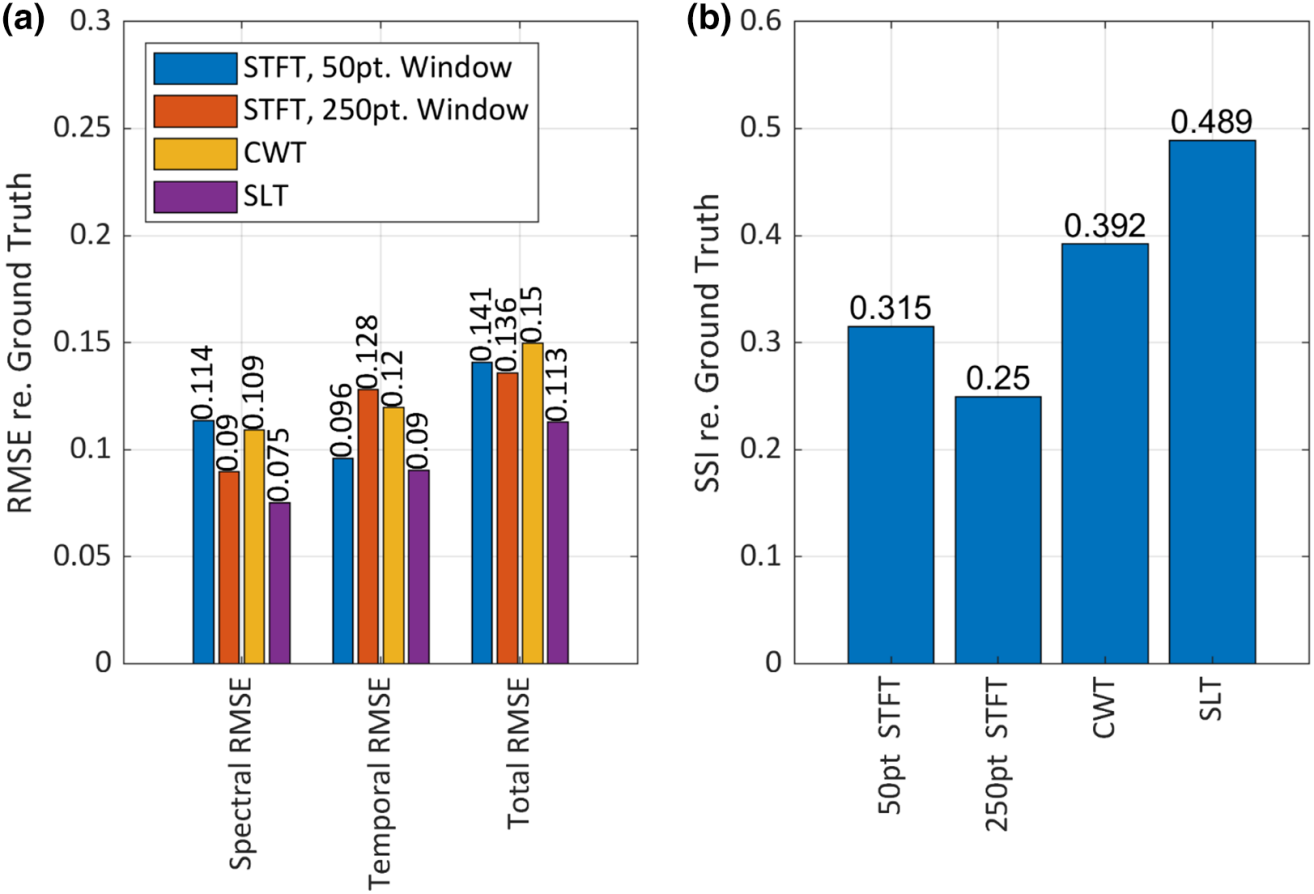
(a) Root mean square error (RMSE) between each algorithmic time–frequency representation (TFR) and the ground truth TFR. In addition to the matrix RMSE (total error), results are also given for the mean RMSE of every row (spectral error) and every column (temporal error). Lower error values indicate better agreement with ground truth. (b) Structural similarity index (SSI) between each algorithmic TFR and the ground truth TFR. Higher SSI scores indicate better agreement with ground truth.

In reference to the ground truth time–frequency representation (TFR), the 50‐point short‐time Fourier transform (short STFT) showed higher root mean square error (RMSE) than the 250‐point STFT (long‐STFT) in terms of frequency (spectral RMSE_short‐STFT_ = 0.114 and spectral RMSE_long‐STFT_ = 0.09, Figure 9a), but lower error in terms of time (temporal RMSE_short‐STFT_ = 0.096 and temporal RMSE_long‐STFT_ = 0.128, Figure 9a). The deltas in RMSE scores between short‐STFT and long‐STFT were similar for the temporal and spectral metrics (spectral RMSE_Δshort‐STFT, long‐STFT_ = 0.024 and temporal RMSE_Δshort‐STFT, long‐STFT_ = 0.023, Figure 9a) which indicates that the two STFTs were biased by equal measures, one towards temporal detail and the other towards spectral detail. The continuous wavelet transform (CWT) scored in‐between the two STFTs for the spectral and temporal error measures (spectral RMSE_CWT_ = 0.109 and temporal RMSE_CWT_ = 0.12, Figure 9a), and showed slightly higher error than both STFTs for the total error measure (total RMSE_short‐STFT_ = 0.141, total RMSE_long‐STFT_ = 0.136 and total RMSE_CWT_ = 0.15, Figure 9a) The superlet transform (SLT) produced TFRs with the lowest error across all measures (spectral RMSE_SLT_ = 0.075, temporal RMSE_SLT_ = 0.09 and total RMSE_SLT_ = 0.113, Figure 9a), outperforming the next best method in spectral RMSE by a margin of 0.15, temporal RMSE by 0.006 and total RMSE by 0.023 (Figure 9a). In terms of percentage differences in total RMSE, the SLT was 18.48% more accurate than the next best‐performing algorithm, the long‐STFT and 28.08% more accurate than the worst performer, the CWT.

The SLT scored the highest on the structural similarity index (SSI, re. ground truth), (SSI_SLT_ = 0.489, Figure 9b). The next highest score was the CWT (SSI_CWT_ = 0.392, Figure 9b), followed by the short STFT (SSI_short‐STFT_ = 0.315, Figure 9b) and long STFT (SSI_long‐STFT_ = 0.25, Figure 9b) The percentage difference in SSI between the SLT and the next best performer (the CWT) was 21.95%, and 64.81% between the SLT and the worst performer, the 250 pt STFT.

### 3.2 Qualitative evaluation – synthetic test signal

**FIGURE 10:**
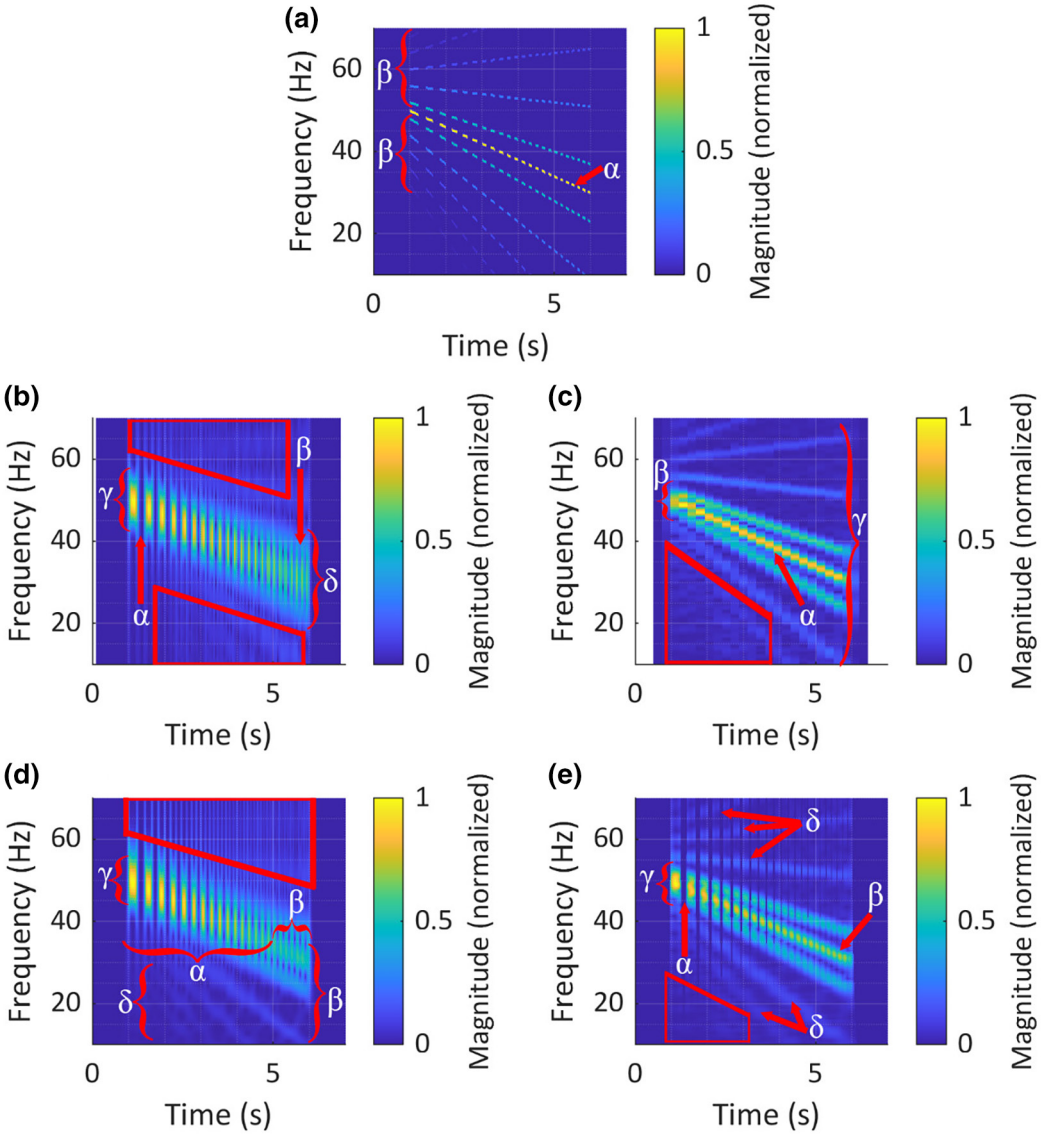
(a) Ground truth time–frequency representation (TFR) of the test signal. (b) 50‐pt STFT spectrogram (short‐STFT) of test signal; overlap = 75%, nFFT = 1250, *fs* = 250 Hz and window shape = Hann. (c) 250‐pt STFT spectrogram (long‐STFT) of test signal; overlap = 75%, nFFT = 1250, *fs* = 250 Hz and window shape = Hann. (d) CWT scalogram of test signal; *Fs* = 250 Hz, time bandwidth product = 200, wavelet type = symmetric, analytic morse. (e) SLT scalogram of test signal, *Fs* = 250, initial number of cycles in superlet = 3 and interval of superresolution orders = [10, 40], multiplicative superresolution. An ideal TFR bears maximum resemblance to the ground truth TFR.

In the short‐STFT TFR, temporal resolution was sufficiently high that the AMs around time = 1 (*s*) were clearly visible as silent periods between pulses (‘α’; Figure 10b), although some temporal smearing did occur towards the end of the signal, where the modulation rate increased (‘β’; Figure 10b). Frequency resolution was not sufficient to accurately visualise the signal's harmonic structure, although the downward FM did appear smooth and continuous. At time = 1 (*s*), discrete components were not distinguishable, but rather appeared as a single broadband pulse (‘γ’; Figure 10b). At time = 6 (*s*), where the components were further spaced in frequency, higher‐order sideband components were somewhat visible, but frequency smearing rendered low‐order sideband components and the carrier component together as broadband pulses (‘δ’; Figure 10b). Frequency smearing between low‐magnitude sideband components resulted in spurious noise across the TFR (red boxes, Figure 10b). Despite the reasonable rendering of AM, this TFR was judged subjectively to bear little resemblance to the ground truth TFR (Figure 10b, re. A).

The long STFT did not have sufficient time resolution to render AMs at any time index; silent periods between pulses were not visible (Figure 10c). Frequency resolution was sufficiently high to accurately visualise the signal's harmonic structure, and the smooth downward FM in the carrier tone appeared discontinuous and stepped (‘α’; Figure 10c). At time = 1 (*s*), the carrier signal was blurred together with the first upper and first lower sideband components (‘β’; Figure 10c), although higher in frequency, distinct components were visible. From time ≈ 2.2 (*s*) continuing to time = 6 (*s*), all sideband components were clearly distinguishable (‘γ’; Figure 10c). Temporal smearing between low‐magnitude, low‐frequency sideband components resulted in spurious noise in the lower half of the TFR (red box, Figure 10c) This TFR was judged to bear reasonable resemblance to the ground truth TFR, but only in terms of harmonic structure (Figure 10c, re. A).

The CWT performed well in terms of temporal resolution at higher frequencies, rendering AMs above ≈35 Hz clearly at time = 1 (*s*) until time ≈ 5 (*s*) with silent periods between pulses were clearly visible during this region of the TFR (‘α’ Figure 10d). Temporal smearing increased with decreasing frequency, obscuring AMs below ≈38 Hz, especially beyond time ≈ 5 (*s*) (‘β’; Figure 10d). Frequency resolution was not sufficient to accurately visualise the signal's harmonic structure, although FM was smooth and continuous. At time = 1 (*s*), all components were blurred together (‘γ’; Figure 10d); although below ≈30 Hz and after time ≈ 2 (*s*), distinct components were faintly visible (‘δ’; Figure 10d). Frequency smearing between low‐magnitude, high‐frequency sideband components resulted in spurious noise in the upper half of the TFR (red box, Figure 10d). Despite good visualisation of AM at high frequencies, spectral details were only resolved at very low frequencies, and overall, the CWT TFR was judged to bear little reasonable resemblance to the ground truth TFR (Figure 10d, re. A).

The TFR produced by the SLT featured sufficient temporal resolution to clearly visualise AMs at time = 1 (*s*), and silent periods between pulses were well defined (‘α’; Figure 10e). At time = 6 (*s*), AM remained visible, although silent periods were less clearly defined (‘β’; Figure 10e). Frequency resolution was sufficient to accurately visualise harmonic structure and FM was smooth and continuous. At time = 1 (*s*), the carrier signal was blurred together with the first upper and lower sideband components (‘γ’; Figure 10e), although higher‐order sidebands were rendered as distinct components (‘δ’; Figure 10e). At time = 6 (*s*), sideband components remained visible. Minimal spurious noise was present in among the fourth‐, fifth‐ and sixth‐order lower sidebands (red box, Figure 10e). This TFR was judged to bear strong resemblance to the ground truth TFR (Figure 10, re. A).

Overall, the SLT was subjectively judged to be most similar to the ground truth, followed by the long STFT, CWT and the short STFT.

### 3.3 Animal sounds

**FIGURE 11:**
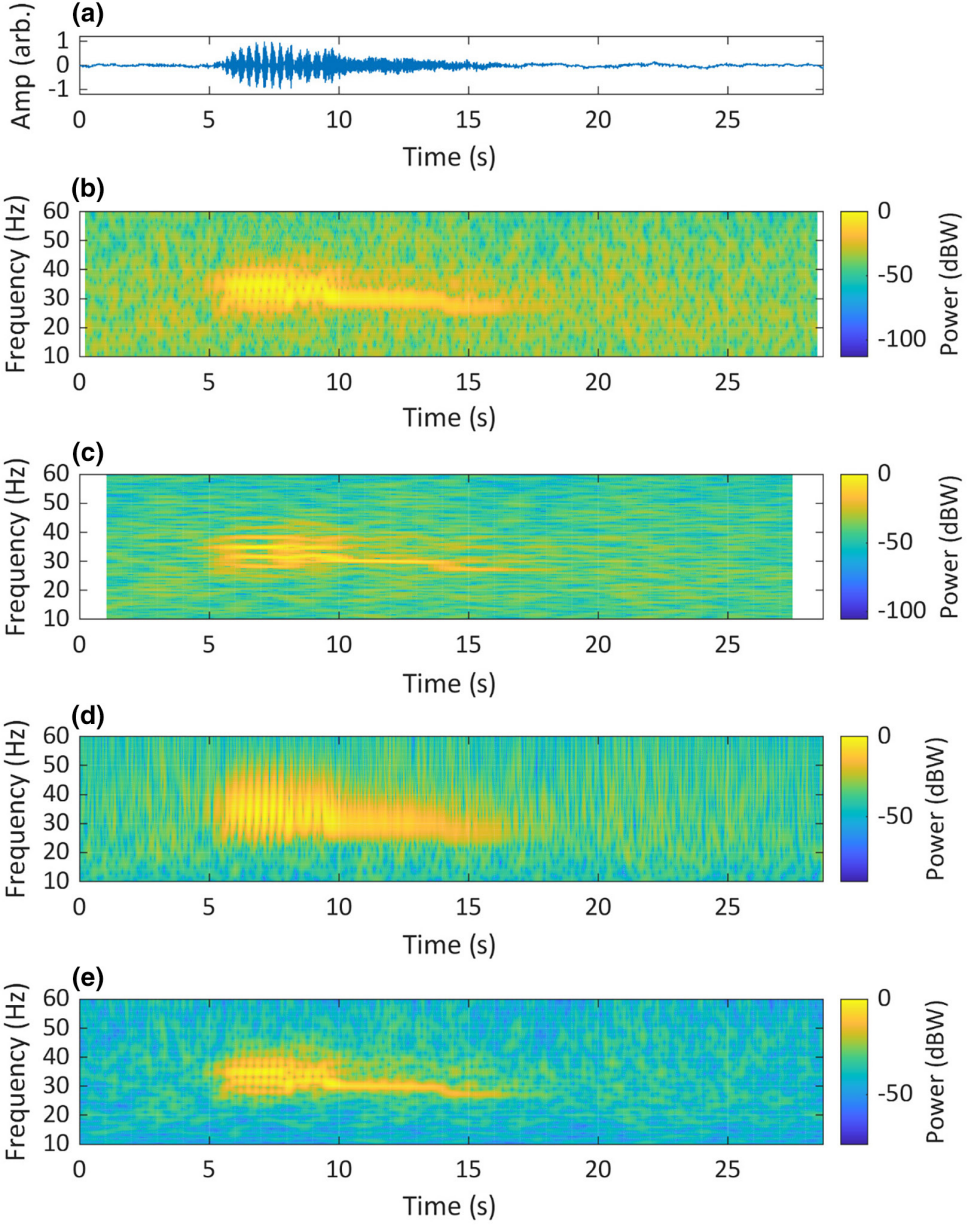
A recording of the Chagos pygmy blue whale (*Balaenoptera musculus brevicauda*) song, originally recorded at 250 Hz. The recording is visualised as: (a) a time‐domain waveform; (b) a short‐STFT spectrogram *Fs* = 120 Hz, *n* = 50, overlap = 90% and nFFT = 1200; (c) a long‐STFT spectrogram *Fs* = 120 Hz, *n* = 250, overlap = 90% and nFFT = 1200; (d) a CWT scalogram, time‐bandwidth product = 60 and voices per octave = 48; (e) an SLT scalogram, initial superlet cycles = 4, multiplicative superresolution order interval = 10:40, frequency range = 10–60 Hz and frequency resolution = 0.1 Hz. Recording taken at the Chagos Archipelago, Indian Ocean, isolated by Dr Emmanuelle Leroy from the CTBTO's IMS hydrophone dataset, used with permission.

**FIGURE 12:**
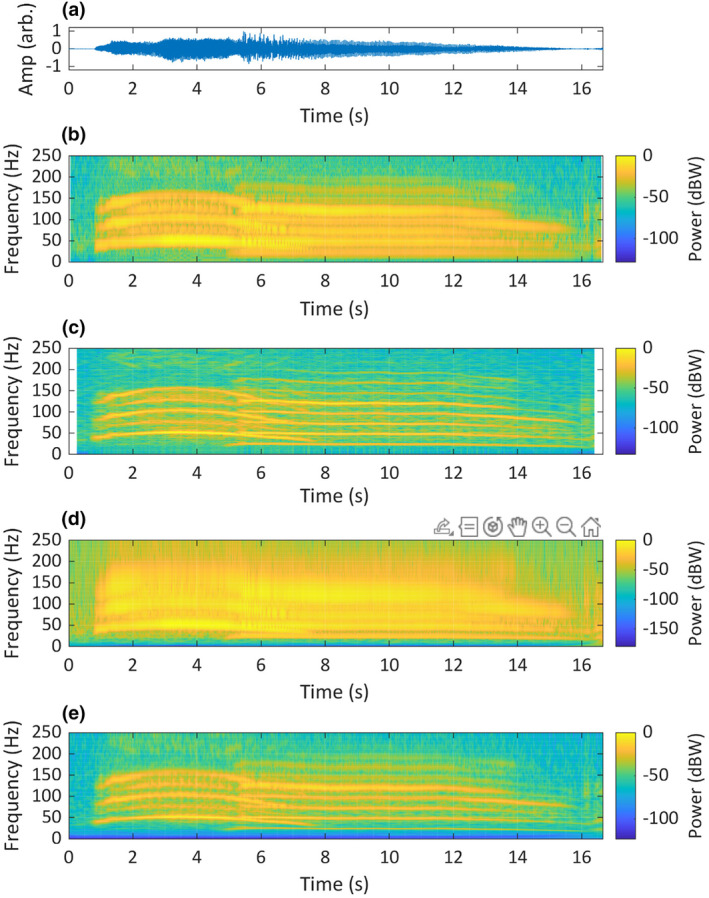
A recording of two consecutive Asian elephant (*Elephas maximus*) rumbles, produced by two individuals, originally recorded at 48 kHz. The recording is visualised as: (a) a time‐domain waveform; (b) a short‐STFT spectrogram, *Fs* = 500 Hz, *n* = 50, overlap = 90% and nFFT = 5000; (c) a long‐STFT spectrogram, *Fs* = 500 Hz, *n* = 250, overlap = 90% and nFFT = 5000; (d) a CWT scalogram, time‐bandwidth product = 60 and voices per octave = 48; (e) an SLT scalogram, initial superlet cycles = 4, multiplicative superresolution order interval = 10:40, frequency range = 0–250 Hz and frequency resolution = 0.1 Hz. Recording provided by Marc Anderson of Wild Ambience used with permission.

**FIGURE 13:**
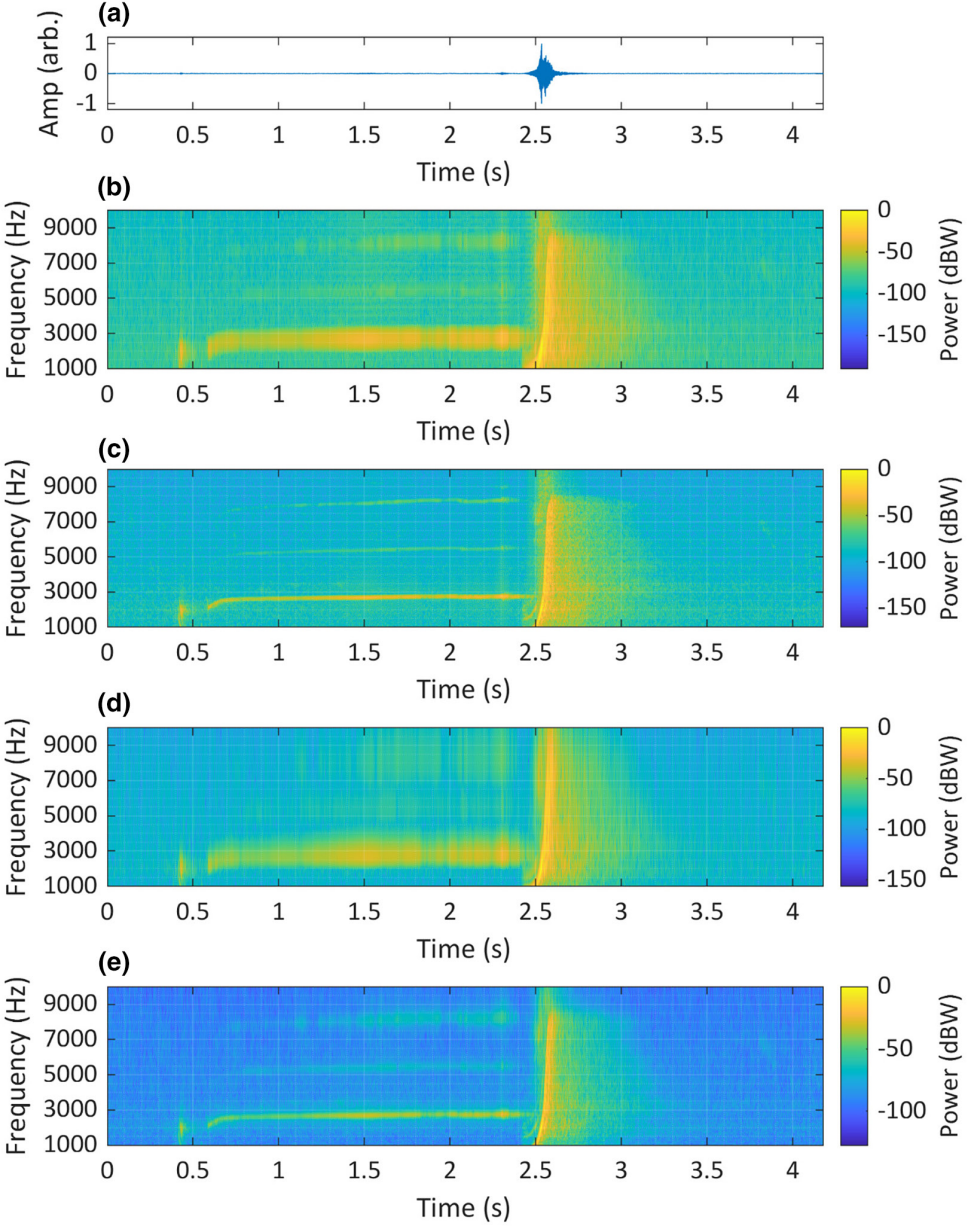
A recording of an eastern whipbird (*Psophodes olivaceus*) song, originally recorded at 48 kHz. The recording is visualised as: (a) a time‐domain waveform; (b) a short‐STFT spectrogram, *Fs* = 20 kHz, *n* = 50, overlap = 90% and nFFT = 10,000; (c) a long‐STFT spectrogram, *Fs* = 20 kHz, *n* = 250, overlap = 90% and nFFT = 10,000; (d) a CWT scalogram, time‐bandwidth product = 60 and voices per octave = 48; (e) an SLT scalogram, initial superlet cycles = 4, multiplicative superresolution order interval = 10:40, frequency range = 1–10 kHz and frequency resolution = 2 Hz. Recording is an excerpt taken from a longer recording, ‘Psophodes olivaceus (ML557908221)’, by David Secomb at Cardinia, Victoria, Australia, courtesy of The Macaulay Library at the Cornell Lab of Ornithology, and was used with permission.

**FIGURE 14:**
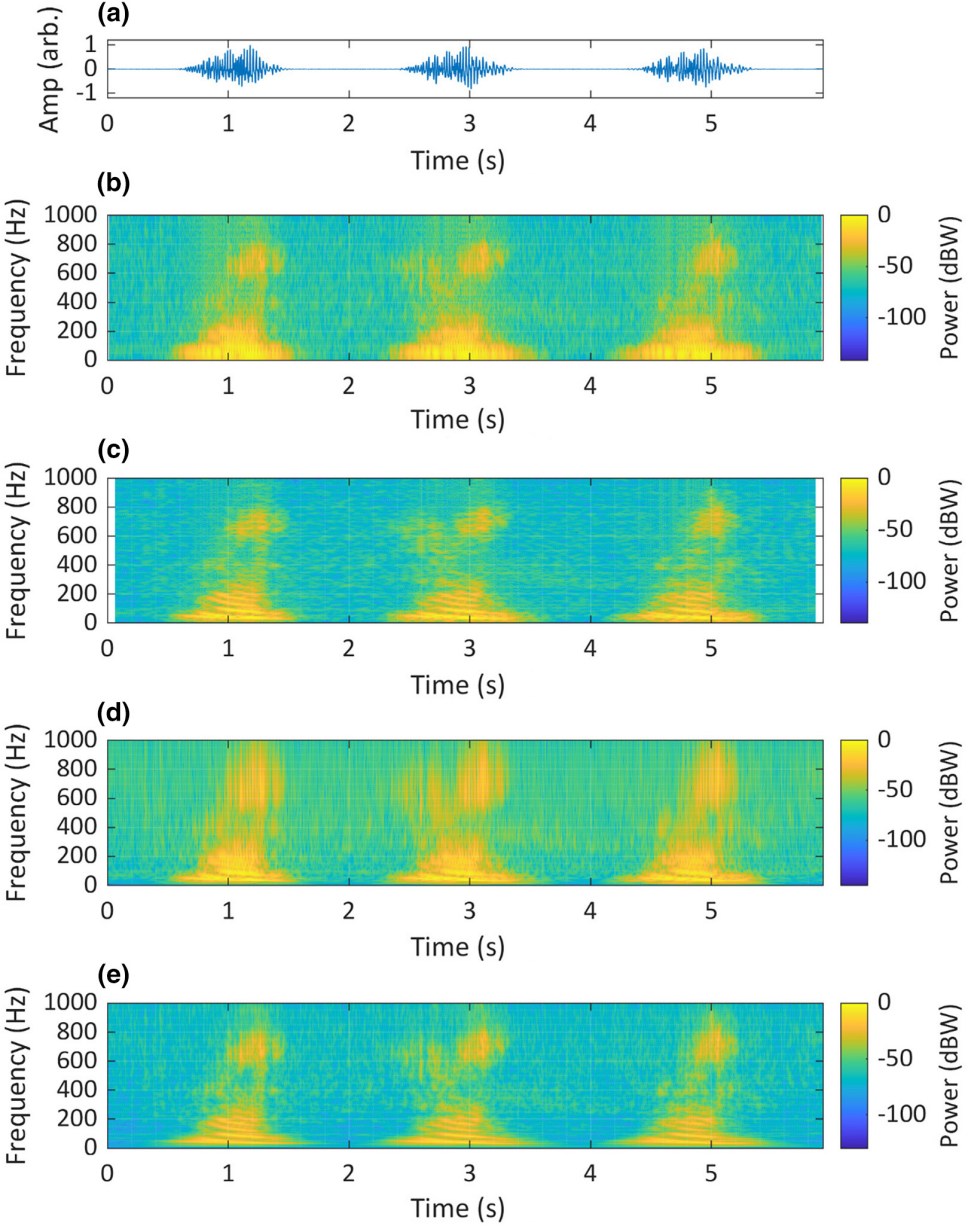
A recording of a southern cassowary (*Casuarius casuarius*) grunt, originally recorded at 48 kHz. The recording is visualised as: (a) a time‐domain waveform; (b) a short‐STFT spectrogram, *Fs* = 2 kHz, *n* = 50, overlap = 90% and nFFT = 20,000; (c) a long‐STFT spectrogram, *Fs* = 2 kHz, *n* = 250, overlap = 90% and nFFT = 20,000; (d) a CWT scalogram, time‐bandwidth product = 60 and voices per octave = 48; (e) an SLT scalogram, initial superlet cycles = 4, multiplicative superresolution order interval = 10:40, frequency range = 0 Hz to 1 kHz and frequency resolution = 0.1 Hz. Recording provided by Marc Anderson of Wild Ambience used with permission.

**FIGURE 15:**
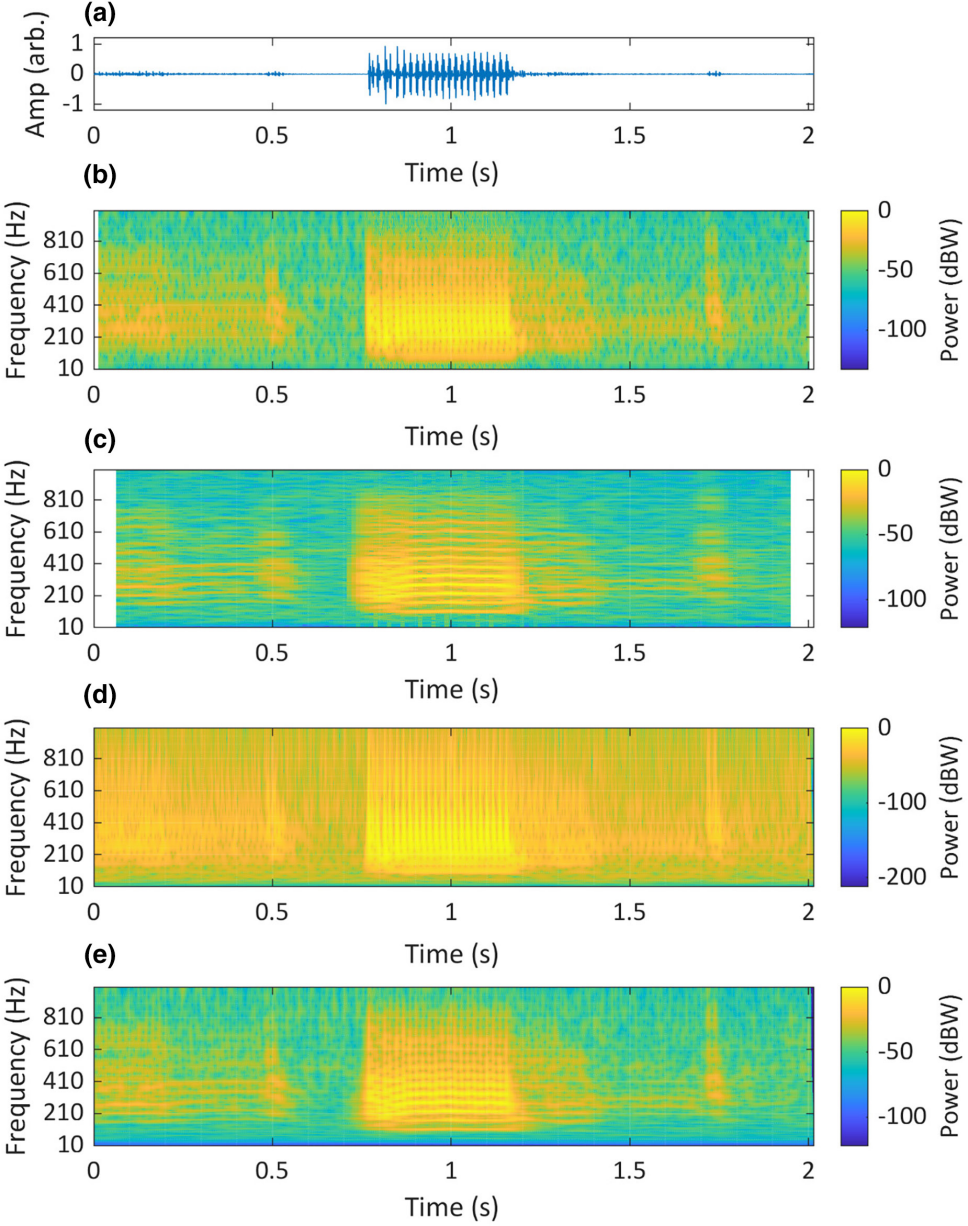
A recording of a mulloway (*Argyrosomus japonicus*) grunt, originally recorded at 5208 Hz. The recording is visualised as: (a) a time‐domain waveform; (b) a short‐STFT spectrogram, *Fs* = 2 kHz, *n* = 50, overlap = 90% and nFFT = 20,000; (c) a long‐STFT spectrogram, *Fs* = 2 kHz, *n* = 250, overlap = 90% and nFFT= 20,000; (d) a CWT scalogram, time‐bandwidth product = 60 and voices per octave = 48; (e) an SLT scalogram, initial superlet cycles = 4, multiplicative superresolution order interval = 10:40, frequency range = 10 Hz to 1 kHz and frequency resolution = 0.1 Hz. Original recording made at Swan River, Western Australia (Parsons et al., 2013), sourced from fishsounds.com (Looby et al., 2023).

**FIGURE 16:**
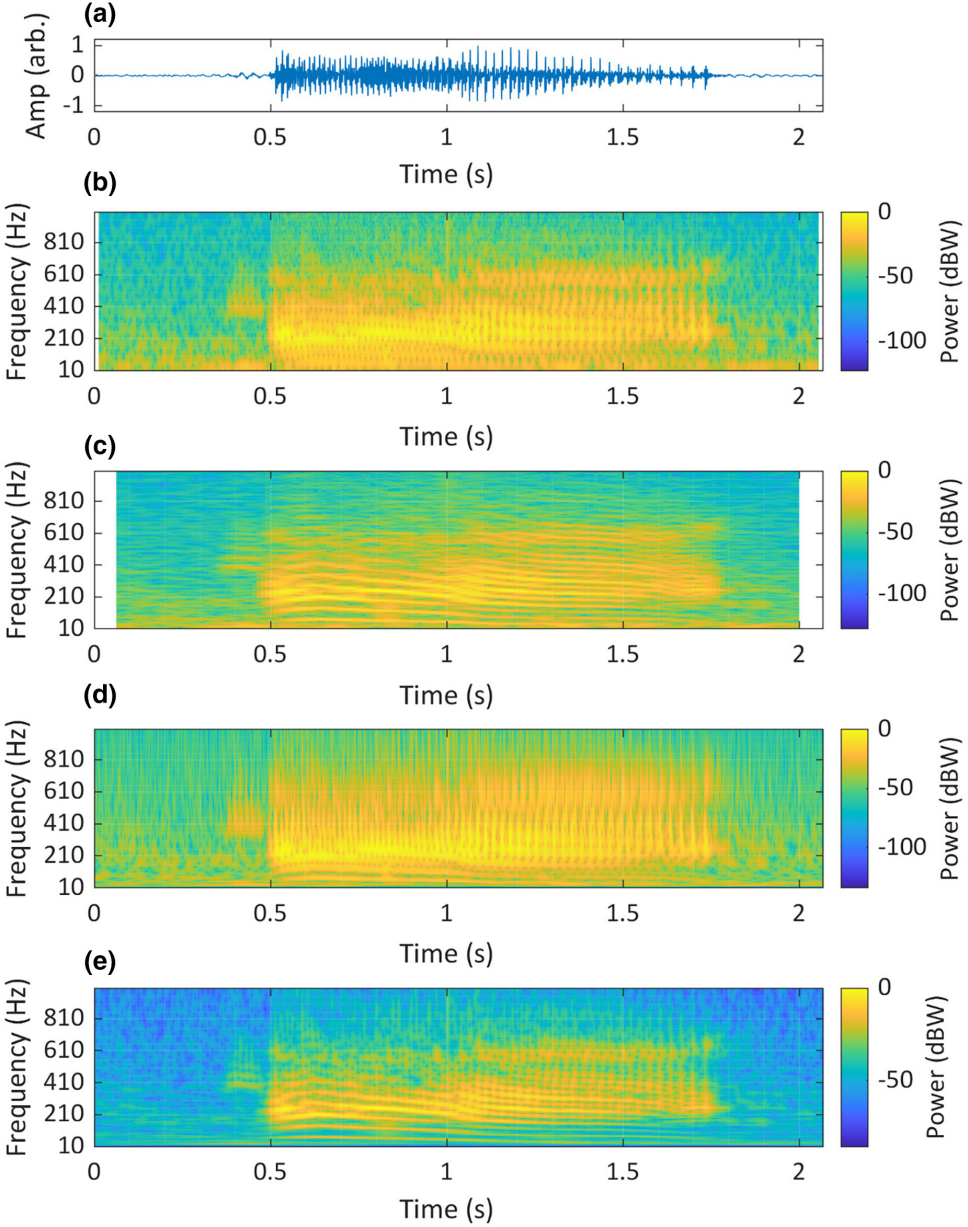
A recording of an American crocodile (*Crocodylus Acutus*) mating growl, originally recorded at 16 kHz. The recording is visualised as: (a) a time‐domain waveform; (b) a short‐STFT spectrogram, *Fs* = 2 kHz, *n* = 50, overlap = 90% and nFFT= 20,000; (c) a long‐STFT spectrogram, *Fs* = 2 kHz, *n* = 250, overlap = 90% and nFFT = 20,000; (d) a CWT scalogram, time‐bandwidth product = 60 and voices per octave = 48; (e) an SLT scalogram, initial superlet cycles = 4, multiplicative superresolution order interval = 10:40, frequency range = 10 Hz to 1 kHz and frequency resolution = 0.1 Hz. Original recording was provided by Benko and Perc (2009) and was used with permission.

To further illustrate the relative performance of the time–frequency analysis methods evaluated above, we present visualisations of six animal sounds, as time‐domain waveforms, two STFT spectrograms (one biased towards frequency resolution and the other towards time resolution), a continuous wavelet transform scalogram and a superlet transform scalogram. These results represent a range of vertebrate taxa: the Chagos pygmy blue whale (Figure 11); the Asian elephant (Figure 12); the eastern whipbird (Figure 13); the southern cassowary (Figure 14); the mulloway (Figure 15) and the American crocodile (Figure 16).

## 4 DISCUSSION

In our evaluation of time–frequency analysis methods using a synthetic test tone, the SLT was shown to be the optimal algorithm. By all objective measures, the SLT showed the best agreement with ground truth. The SLT was also subjectively judged to be the best‐performing method of those tested for visualising complex, low‐frequency animal sounds. The two short‐time Fourier transform (STFT) methods performed well in the domain they were biased towards but failed across all tests to visualise both temporal and spectral details simultaneously. The wavelet convolution technique underlying the continuous wavelet transform (CWT) (Mallat, 1999) offers a compromise between time and frequency resolution. The results here show, however, that the compromise ultimately proves ineffectual, producing visualisations that are biased towards time resolution. The CWT's, time and frequency resolution are determined primarily by the number of wavelet cycles (Moca et al., 2021), and the SLT operates by taking the geometric mean of a plurality of CWTs, each having differing numbers of wavelet cycles (Moca et al., 2021). It is therefore not surprising that the SLT produces visualisations that achieve that time–frequency resolution compromise better than the CWT.

The second‐best and worst performers were reversed comparing the SSI and total RMSE measures, with the CWT performing second in SSI, but last in total RMSE, and long STFT last in SSI and second best in total RMSE. This at first appears to indicate a difference in how these measures weigh the importance of temporal versus spectral details, since the CWT biases towards time resolution and long STFT towards frequency. More likely though is that this difference indicates the influence of structure on the measures. Where RMSE measures error between images pixel for pixel, SSI is built on a model of human vision and measures similarity in image structures by considering clusters of pixels as perceptually inter‐dependent (Wang et al., 2004). Despite SSI's intention to correlate with human perception, the qualitative evaluations tend towards agreement with the RMSE results. The RMSE may therefore be more conclusive than SSI, but in any case, both measures show the SLT to be the most accurate visualisation method for the test signal.

In the qualitative evaluation using the same synthetic test signal, the SLT TFR was judged to bear the greatest resemblance to the ground truth TFR. The short‐STFT performed well in time resolution, but poorly in frequency resolution, and for the long‐STFT, the inverse was true. In the CWT TFR, temporal resolution was high, but frequency resolution degraded towards the top of the analysis bandwidth, and cross‐term artefacts negatively impacted readability. The SLT was judged to be the best‐performing method of those tested for the visualisation of the synthetic test signal.

In five of the six comparisons of TF analysis methods using recorded animal sounds, the SLT visualisations represented a compelling compromise between time and frequency resolution, while the other methods tended towards TF bias. The SLT visualisation of the Chagos pygmy blue whale song (Figure 11) bore the strongest resemblance to the descriptions by Sousa and Harris (2015) and Leroy et al. (Leroy et al., 2021), including amplitude modulations (AM) with the same pulse rate identified by Leroy et al. (2021). The visualisations produced with the other methods all had insufficient resolution in either frequency or time and could not be said to wholly match the established description of the song.

The visualisation of the two Asian elephant calls (Figure 12) shows acoustic features not agreed upon in the literature. Nair et al. (2009) classified these calls as ‘rumbles’. Based on observations by Beeck et al. (2022), the first call may be a sub‐type of rumble that occurs during agitated greetings, described as having a higher frequency and stronger frequency modulation than other rumbles. Asian elephant vocalisations were originally described as pulsed call types termed ‘growls’, ‘rumbles’, ‘roars’ and ‘motorcycles’ (McKay, 1973), however, the spectrograms used in that study had insufficient detail to show pulsing. Subsequent descriptions of rumbles (Beeck et al., 2022; de Silva, 2010; Garstang, 2004; Leong et al., 2003; Nair et al., 2009; Payne et al., 1986) are of tonal and harmonic calls, with no mention of AM or pulsing. The STFT spectrograms in those studies were biased towards frequency resolution. Our SLT rendering of the ‘agitated greeting rumble’ in Figure 12 may show AM in the overtone components, also visible, although less clearly, in the 50 pt STFT. The SLT appears to show the pulsing described by McKay (1973), suggesting that the use of STFT spectrograms with insufficient temporal detail may have led to misinterpretation of this call in more recent literature.

In the case of the eastern whipbird (Figure 13), other than a modest improvement in background noise suppression, there did not appear to be significant advantage to the SLT. Indeed, the frequency resolution in the 250‐point STFT was better than in the SLT. This was expected, as this sound lies across the widest and highest bandwidth of all those analysed (approx. 1 to 10 kHz), and therefore represents the greatest challenge in terms of visualising harmonic structure with high resolution. Additionally, this animal sound is a simple, tonal sound, so there are no complex temporal details for the SLT to reveal that might have been otherwise hidden.

Our SLT visualisation of the southern cassowary call (Figure 14) shows greater structure than previously described. Mack and Jones (2003) describe this call as a sequence of large‐scale pulses between 32 Hz and 50 Hz, with no mention of smaller‐scale AM (or pulsing) within the large‐scale pulses. Mack & Jones' spectrogram (2003) has insufficient resolution to see small‐scale pulses. Our SLT visualisation of the southern cassowary shows small‐scale pulsing between 600 Hz and 800 Hz, which Mack and Jones (2003) do not describe. This may be because their spectrogram does not show frequencies above 100 Hz. Our visualisation of the cassowary call may show previously undescribed acoustic features, although analysis of many more calls would be necessary to determine this.

Parsons et al. (2013) describe the mulloway call as amplitude modulated, with a pulse rate of 59 Hz, although this structure is not evident in the STFT spectrograms they provide, and it is shown instead via time‐domain waveforms. The pulses are clearly visible in our SLT, short‐STFT and CWT visualisations, but not in our long‐STFT (Figure 15), where all temporal details are lost. The CWT has severe spectral leakage (i.e. where new frequency components are created), and individual frequency components are not discernible, except for the lowest four. Our short‐STFT also has severe spectral leakage, although distinct components are visible. The SLT shows distinct components, and overall, the structure it shows bears the closest resemblance to the description by Parsons et al. (2013).

The roar of the American crocodile (Figure 16) is not described in detail in the literature, and only one publication (Benko & Perc, 2009) could be found that provided a spectrogram. It does not show any AM. Benko and Perc (2009) describe the sound as non‐linear, with the bulk of the energy occurring between 100 and 300 Hz. Dinets (2013) did not provide visualisations, but described infrasonic components around 10 Hz. Neither description mentions amplitude modulation or pulsing. Our SLT shows both the 100–300 Hz components and the 10 Hz infrasonic components, in addition to amplitude modulations, particularly in the latter part of the call. These AM features are visible in the CWT and short STFT, but not the long STFT. The CWT and short STFT both feature substantial spectral leakage and lose frequency definition.

While the present study did not aim to investigate each TF analysis method's resilience to noise, and no objective tests were included to measure this variable, it is interesting to note that in all six quantitative evaluations with recorded animal sounds, the SLT was subjectively judged to show better background noise suppression. This may be an expected outcome, given that the SLT features less temporal and spectral smearing than other methods, so small, isolated regions of real background noise in the TFRs remained small and isolated. In other methods that are subject to greater temporal and spectral smearing, it stands to reason that any such regions of energy owing to actual noise would be spuriously distributed in time and frequency, creating the false impression of higher background noise levels.

The results suggest that while the SLT is a substantial improvement on conventional TF visualisation methods when analysing complex, low‐frequency animal sounds, there remains no single, best, ‘one size fits all’ approach to TF analysis. In some cases, there may be valid justifications for using the STFT, the CWT or other methods. It is reasonable, for example, to use a long‐windowed STFT to visualise an Antarctic blue whale ‘Z‐call’, due to the fact that it is well documented, and known to consist of a single, steady tone, with no AM or other temporal complexity (McDonald et al., 2006), and therefore lacks any features that a long‐windowed STFT would fail to visualise. For an animal sound that is new or under‐documented, more consideration would be warranted as to the ideal method. The STFT is also faster to compute than the SLT, which for long recordings such as those used in acoustic ecology, maybe a factor to consider. Ideally, every analysis of an animal sound would begin with a rigorous evaluation of the available TF visualisation methods to find the best possible one for each use case and configure it optimally. Due to time constraints, knowledge gaps and insufficient tooling, this kind of deep methodological experimentation in TF analysis of animal sounds may not be feasible or practical. It also does not appear common in bioacoustics to explain why or how particular STFT settings were selected. This suggests it may be common practice to use ‘default’ settings for STFT parameters, rather than to optimise them for each sound under investigation. This is where the SLT has a subtle but important advantage beyond its improved accuracy.

While the SLT does have tuneable parameters, this study found that the parameters used to produce optimal visualisations for one animal sound produced similarly accurate visualisations for all the animal sounds tested, regardless of their character. The lower frequency limit and frequency resolution parameters were tuned for each animal sound, and the sampling frequency of each sound differed, however, no other SLT parameters were altered between the animal sounds in the qualitative analyses. The six sounds differ substantially in their bandwidths, harmonic and temporal structures, and yet, the SLT produced TFRs that were minimally affected by TF bias and were easy to interpret and auralise, with minimal tuning of the algorithm. This apparent insensitivity to signal content may be the SLT algorithm's greatest strength. Indeed, Moca et al. (2021) state in their original description of the SLT, that one of its primary benefits is the simplicity with which it can be implemented, and the lack of expertise required to configure its parametric controls. If indeed it is common practice in bioacoustics to perform TF analysis without rigorous parameter optimisation for each sound analysed, then the SLT algorithm is ideal for adoption by this field, as it can, at least to some extent be used in that manner, while still producing visualisations that are less biased than those produced by conventional methods.

## 5 CONCLUSION

In this study, we compare methods for time–frequency (TF) analysis quantitatively, using a synthetic test signal, and qualitatively using animal sounds. The methods investigated included the conventional method in the biological sciences, the short‐time Fourier transform (STFT), the continuous wavelet transform (CWT) and a recently developed method that generalises and improves upon the CWT, the superlet transform (SLT). Results showed the SLT to be more accurate than the STFT and the CWT in visualising the spectral and temporal character of the test signal as well as five complex, low‐frequency animal sounds. Results also indicate that there is considerable potential for misinterpretation using the STFT and CWT. Perhaps most importantly, the SLT was shown to require minimal parametric tuning, and when optimal parameters were set and held constant, the accuracy of visualisations was consistent for multiple signals that had substantial differences in spectral and temporal structure, and overall bandwidth. Even when considering the SLT's 18.48% to 28.08% reduction in error compared with the CWT and STFT, this ease of use may be the strongest justification for the adoption of the SLT for visualising complex, low‐frequency animal sounds.

## AUTHOR CONTRIBUTIONS

**Benjamin A. Jancovich:** Conceptualization (lead); data curation (lead); formal analysis (lead); investigation (lead); methodology (lead); project administration (lead); resources (lead); software (lead); validation (equal); visualization (lead); writing – original draft (lead); writing – review and editing (equal). **Tracey L. Rogers:** Conceptualization (supporting); data curation (supporting); formal analysis (supporting); investigation (supporting); methodology (supporting); supervision (lead); validation (equal); writing – review and editing (equal).

## CONFLICT OF INTEREST STATEMENT

The authors declare no conflicts of interest and no relevant commercial relationships.

## Supporting information


Appendix S1.

## Data Availability

This work fulfills the criteria for Open Science Bagdges for data and materials. All results in this work are reproducible using the open source code, data, and materials available at the BASSA Software Github Repository, and the Time Frequency Analysis Methods Comparison GitHub repository.
