# Supplementary material for: BASSA: New software tool reveals hidden details in visualisation of low‐frequency animal sounds
Source: Ecol Evol. 2024 Jul 3;14(7):e11636. doi: 10.1002/ece3.11636 (PMC11220835; doi:10.1002/ece3.11636)
Supplement: Supplementary file 1 — Appendix S1. [file ECE3-14-e11636-s001.docx]

BASSA: new software tool reveals hidden details in visualisation of low frequency animal sounds.

Supplementary Material

Benjamin A. Jancovich^1*^ & Tracey L. Rogers^2^
Centre for Marine Science & Innovation
School of Biological, Earth and Environmental Sciences
Room 5107, Level 5 West Biological Sciences South (E26)
University of New South Wales, Kensington, 2052, NSW, Australia

^1^ [
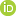
](https://orcid.org/0000-0003-3592-787X) [0000-0003-3592-787X](https://orcid.org/0000-0003-3592-787X)
^2^ [
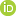
](https://orcid.org/0000-0003-3592-787X) 0000-0002-7141-4177
* Correspondence to [✉](mailto:b.jancovich@unsw.edu.au) [b.jancovich@unsw.edu.au](mailto:b.jancovich@unsw.edu.au)

**Table of Contents**

[1 A Review of time-frequency analysis methods 3](#_Toc139894970)

[2 Quantitative Evaluation Methods 5](#_Toc139894971)

[2.1 Test Signal Synthesis 5](#_Toc139894972)

[2.2 Ground Truth 7](#_Toc139894973)

[2.3 Performance Measures 9](#_Toc139894974)

[3 Introducing the BASSA Software Application 12](#_Toc139894975)

[3.1 Software Interface 13](#_Toc139894976)

[3.1.1 The Pre-Processing Tab 13](#_Toc139894977)

[3.1.2 The Audio Playback Panel 14](#_Toc139894978)

[3.1.3 The Superlet Scalogram Tab 15](#_Toc139894979)

[3.2 Limitations 18](#_Toc139894980)

[3.3 Performance Benchmarks 19](#_Toc139894981)

[3.4 Future Development 20](#_Toc139894982)

[4 References 20](#_Toc139894983)

# A Review of time-frequency analysis methods

Here we discuss several commonly used methods for time frequency (TF) analysis that have been developed since the original and most widely used method, the short-time Fourier transform (STFT) spectrogram. The most common of these is a variant of the STFT spectrogram called the reassigned spectrogram. This method aims to sharpen blurry time-frequency data by relocating the energy according to local estimates of instantaneous frequency and group delay, and this method can be applied to a number of TF analysis algorithms ​(Auger & Flandrin, 1995). The main limitation of spectrogram reassignment is that in the presence of high levels of background noise, the reassignment can introduce artifacts. This is also the case when the spectral components in the signal of interest are overlapping or closely spaced in time or frequency (Averbuch, 2021). While the standard STFT is the most common method used in bioacoustics, it is not clear how common reassignment is, as its use is seldom reported alongside other STFT parameters.

The Wigner-Ville distribution function (WVDF) (S. G. Mallat, 1999, p. 4), uses auto-correlation and the Fourier transform to perform TF analysis with high resolution in both time and frequency. It does have limitations however, most notable of which is cross-term contamination. Cross terms are artifacts that appear when the signal contains multiple components, leading to interference patterns in the time-frequency representation (Gómez et al., 2011). This is problematic in the study of animal sounds, which often contain multiple overlapping or closely spaced frequency components. Variants of the WVDF exist that aim to overcome these limitations using smoothing kernels, all of which come with various trade-offs. These include the Choi-Williams distribution function (CWDF) (Choi & Williams, 1989), which uses an exponential kernel to suppress cross-terms. The unintended side effect of this is suppression of real signal components (Papandreou & Boudreaux-Bartels, 1993), and this forces the user to compromise between eliminating spurious artifacts and retaining details of the real signal.

The phasegram is a novel method for visualising the behaviour of non-linear systems. It applies the spectrogram’s sliding-window concept to phase-space visualisations used in non-linear dynamics to provide an illustration of changes in the structure and character of vibratory modes, as a function of time (Herbst et al., 2013). This method allows for the identification and analysis of non-linear behaviours such as bifurcations (I.e. sudden and drastic changes in the behaviour of a system when a parameter is varied, e.g., a stable periodic vibration transitions to chaotic, unstable vibration as the energy input to the system is increased. See Cazau et al., 2016 for discussion of this phenomenon in animal vocalisation). When these acoustic features tend towards deterministic chaos (noise-like, non-linear sounds that are not truly random), the phasegram reveals structure within the pseudo-noise. Visualisations produced with other methods cannot show this structure and may therefore lead to misinterpretation. In the study of vocal production and phonation, the ability to distinguish between deterministic chaos and truly random noise is important, as each of these acoustic features suggests a different physical vocal production mechanism. The main limitation of the phasegram is that it does not convey absolute frequency or amplitude information, so while it represents a useful tool for investigating non-linear phenomena, it represents a complementary addition to conventional time-frequency visualisation, rather than an alternative. Further, compared with intuitive properties like frequency and amplitude, phase is somewhat more abstract, so the phasegram may be difficult to interpret to users and readers in the fields of biology and ecology.

The multitaper spectrum (Xiao & Flandrin, 2007) is a TF analysis based on Thomson's multitaper analysis method, which in the simplest case, involves taking the mean of multiple STFTs made with a variety of window functions, referred to as tapers. While this method does provide TF representations with reduced spectral curvature bias and less spectral leakage, it requires the user to select an optimal set of tapers, which introduces complexity and increases the expertise required to use effectively. Additionally, the averaging used to suppress random variability in this method can introduce bias (Prieto et al., 2007).

The Multiple Window Savitzky-Golay Filter is a method for enhancing STFT spectrograms, and has been used in analysis of bird song field recordings (Koluguri et al., 2017). This method uses a plurality of smoothing filters to suppress background noise in a spectrogram, and while Koluguri et al., (2017) have shown that it can improve the performance of spectrogram-based automated call detectors, it has not been shown to improve time-frequency resolution, nor is this its intended purpose.

The continuous wavelet transform (CWT) operates differently to the STFT and the above methods. It does not require use of window functions or Fourier transforms, and attempts to resolve the TF bias by prioritising time resolution at the top of the spectrum, and frequency resolution at the bottom (S. G. Mallat, 1999, pp. 4–6). The CWT has been shown to perform better than optimised variants of both the CWDF and WVDF in the visualisation of complex biomedical signals (Karlsson et al., 2000). Being a multiscale transform, the CWT represents a compromise, with relatively poor time resolution at low frequencies and poor frequency resolution at high frequencies, and this makes it problematic for signals with complex TF structures that span large bandwidths. This limitation applies to other wavelet-based transforms such as the Mallat Scattering Transform (S. Mallat, 2012). This method iteratively computes the average of the moduli of a wavelet transformed signal, and is primarily used for signal classification, recognition, and feature extraction applications due to its ability to generate TF representations that are invariant to various deformations such as translation, rotation, dimensionality reduction, and scale separation. Like the CWT, it compromises time resolution at low frequencies and frequency resolution at high frequencies, and due to the nonlinear operations and iterative data transformations involved in computing each modulus, the results can be difficult to interpret when compared to a conventional spectrogram (Bammer et al., 2019; Czaja & Li, 2019).

# Quantitative Evaluation Methods

## Test Signal Synthesis

The time domain test signal $x(t)$ (Figure 1C) was a frequency and amplitude modulated (AM) sinusoid. $x(t)$ is the product of sinusoidal carrier signal $x_{c}(t)$ (Figure 1A) and square wave modulation signal $x_{m}\left( t \right)$ (Figure 1B). The instantaneous frequency of $x_{c}\left( t \right)$ was linearly swept from 50 to 30 Hz, over a duration of 5 seconds, with an initial phase of 0 degrees as per:

(1)

$$x_{c}\left( t \right)=cos[\varphi_{0}+2\pi\left( \frac{c}{2}t^{2}+f_{0}t \right)]$$

Where $\varphi_{0}$ is the initial phase at time = 0 and $c$ is the sweep rate, given by:

(2)

$$c=\frac{{f_{1}-f}_{0}}{T}$$

Here, $f_{0}$ is the sweep start frequency, $f_{1}$ is the sweep end frequency, and $T$ is the total duration of the sweep.

The instantaneous frequency of AM waveform $x_{m}(t)$ was swept linearly from $f_{{mod}^{0}}=2 Hz$ to $f_{{mod}^{1}}=7 Hz$, over a 5 second duration. This waveform was generated by plugging $f_{{mod}^{0}}$ to $f_{{mod}^{1}}$ into equation (*1*), then setting all samples of the result $>0$ to 1, and samples $<0$ to $0$. The initial phase term in equation (*1*) was set to -90 degrees. One second of silence was inserted at both the start and the end of $x_{c}\left( t \right)$ and $x_{m}(t)$, then they were multiplied together to give $x(t)$.


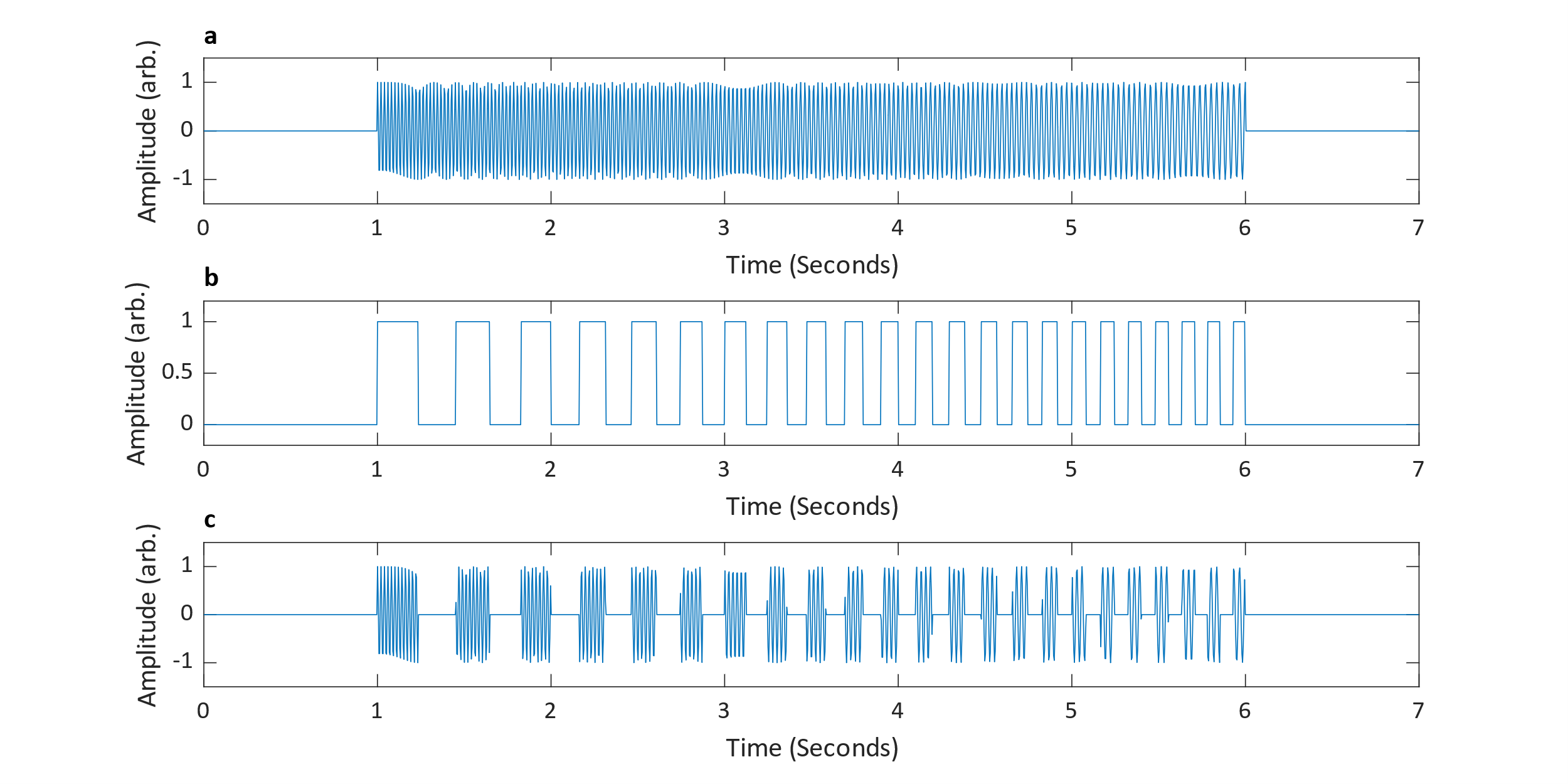


Figure 1: Construction of the synthetic test signal represented in the time domain. Panel A shows the carrier signal x_c (t), panel B shows the amplitude modulation signal x_m (t), and the bottom panel shows the final test signal x(t).

## Ground Truth

Each algorithm returned time-frequency representations (TFRs) as matrix of a different size and aspect ratio. Any rescaling of a TFR that modifies its aspect ratio would distort the proportions of the visualisation, corrupting any evaluation of agreement with ground truth. Therefore, a ground truth TFR was built for each algorithm, matched to the aspect ratio of that algorithm’s output. The content of these TFRs is identical, as is their extent in time and frequency. Only the number of rows and columns differs, corresponding to the differing temporal and spectral resolutions between algorithms.

An oversampling scheme was implemented to avoid aliasing the ground truth due to insufficient number of rows and columns. For algorithmic TFRs with number of rows, $n_{r}$ $<(fmax-fmin)/fres$, or number of columns $n_{c}$ $<fs\cdot2$, the ground truth would be constructed with $n_{r}\cdot p_{r}$ rows, where $p_{r}=\frac{(fmax-fmin)/fres}{n_{r}}$ and with $n_{c}\cdot p_{c}$ columns, where $p_{c}=(fs\cdot2)/n_{c}$. Ground truth matrices therefore had aspect ratios matching their corresponding algorithmic TFRs, upscaled such that they had sufficient sample points to accurately depict the ground truth of the signal’s time-frequency (TF) behaviour.

The ground truth TFR was populated by writing a value of 1 to every element corresponding to the instantaneous frequencies and time indices of $x(t)$, and zeros to all other elements. Sideband components were also included. The starting and ending frequencies for the $i^{th}$ upper sideband component were calculated as:

(3)

$$f_{0{usb}^{i}}=f_{0}+f_{{mod}^{0}} \cdot o$$

(4)

$$f_{1{usb}^{i}}=f_{1}+f_{{mod}^{1}} \cdot o$$

where $o$ is the sideband order, $f_{0}$ and $f_{{mod}^{0}}$ are the starting frequencies of the carrier and modulator signals respectively, and $f_{1}$ and $f_{{mod}^{1}}$ are the ending frequencies of the carrier and modulator signals, respectively. Sweep rates, $c_{{usb}^{i}}$ for the $i^{th}$ sideband components were then computed by plugging $f_{0{usb}^{i}}$and $f_{1{usb}^{i}}$ into eq. (*2*), and the vectors of instantaneous frequencies were derived as per:

(5)

$$f_{{usb}^{1}}\left( t \right)= c_{{usb}^{i}}t+f_{{0usb}^{i}}$$

where $t$ is the time vector. Because $x_{m}(t)$, was a square wave, the $o$ term in eq. (*3*) and (4) was always odd valued$.$ For the lower sidebands, equations (*3*) and (4) were used, but the addition sign was flipped to subtraction. The sideband components were written to the ground truth TFR following the above method for the carrier. Sidebands were magnitude-scaled by half for every increasing order. Matrices for sidebands and the carrier were summed together to produce the ground truth TFR.

To match the frequency resolution of ground truth to the target resolution of 0.2Hz, a column-wise, nearest-neighbour interpolation routine was implemented, where any element in the ground truth containing a value $>0$ was copied to the $\frac{r}{2}$ elements above and below, where $r$ is the ratio of target frequency resolution to actual frequency resolution of the ground truth TFR. Matrix dimensions were not changed.

The AM signal $x_{m}(t)$ was re-synthesised, keeping the same frequency and time values, but with its length in samples matched to the number of columns in the ground truth TFR. This vector was then copied across $n$ rows to match the dimensions of the ground truth TFR, and every element multiplied by every element of the ground truth matrix. The resulting matrix was smoothed with a gaussian image filter using a standard deviation of $\sigma= 0.3$ to produce the final ground truth TFR (Figure 2).


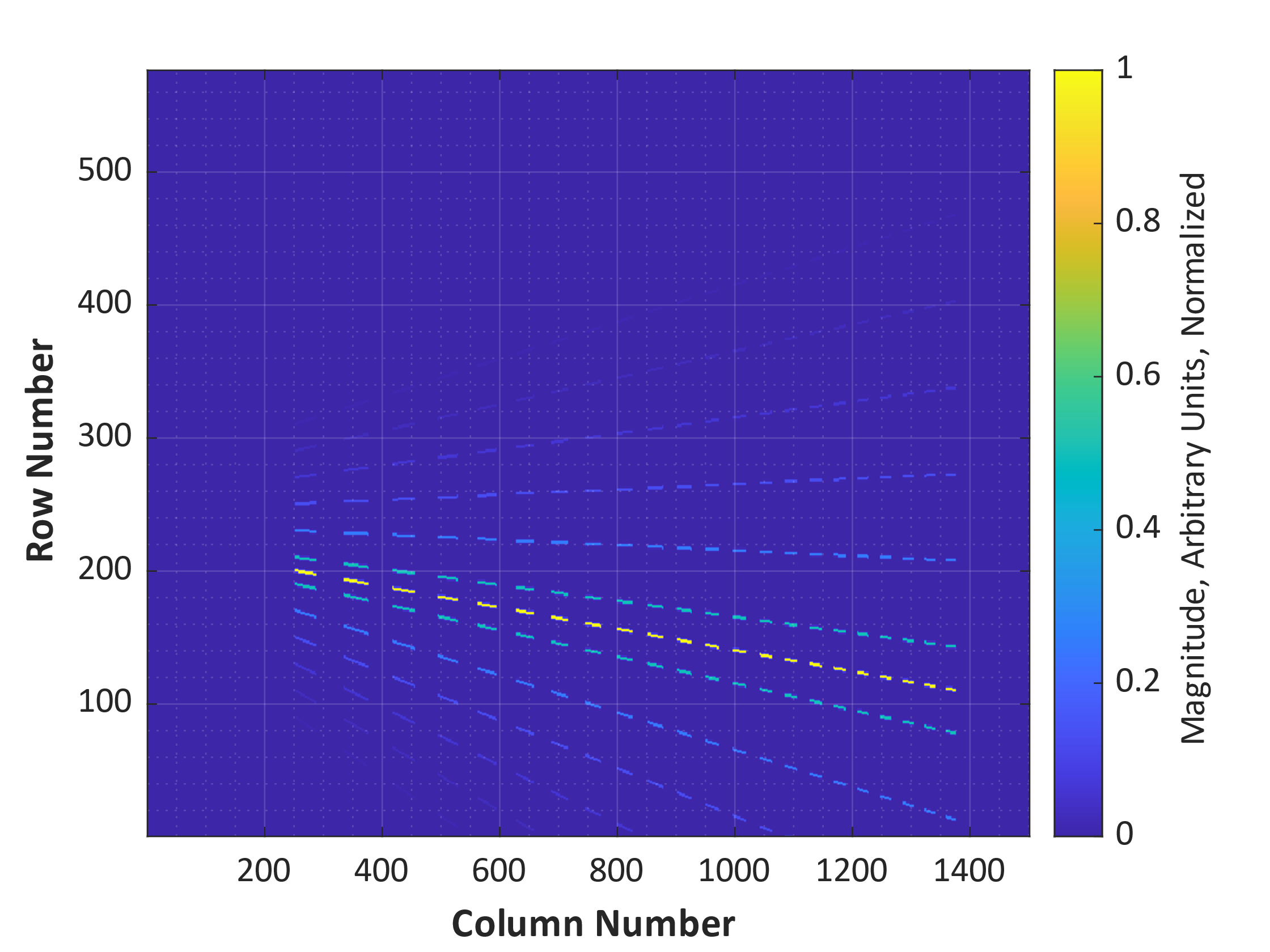


Figure 2: An example ground truth matrix for the carrier signal $x_{c}\left( t \right)$, and the upper and lower sideband components ${usb}^{i}\left( t \right)$ and ${lsb}^{i}(t)$ for $i=[1:6]$. Fs = 250Hz, fmin = 10Hz, fmax = fs/2, f_0_=50, f_1_=30, fmod_0_ = 2, fmod_1_ = 7, fres = 0.2 Hz.

***x_c_(t)***

***usb^i^(t)***

***lsb^i^(t)***

## Performance Measures

The time-frequency representation (TFR) returned by each algorithm was scored on its agreement with its corresponding ground truth matrix. Two measures were used; root mean square error (RMSE), and structural similarity index (SSI). As the matrices were matched in aspect ratio, but not necessarily size, algorithmic TFRs were up-scaled to match the size of their corresponding ground truth TFR using a nearest-neighbour interpolation algorithm.

Three different formulations of RMSE were calculated. First, the RMSE was calculated between the elements of each row of the algorithmic TFRs and Ground Truth TFR, which represented the error for each sampled frequency, then the “Mean Spectral RMSE” was calculated by taking the mean of RMSE for all rows. The same was repeated for each column to derive the “Mean Temporal RMSE”. Third, a “Total RMSE” was calculated, which was the root mean square error between all elements of the algorithmic TFRs and the elements of their corresponding ground truth TFRs.

Structural similarity index (SSI) is a statistical measure of similarity commonly used in image processing and computer vision fields. Structural Similarity Index was calculated by treating the TFR matrices as greyscale images, and following Wang et al. (2004), where for images $x$ and $y$:

(7)

$$SSI\left( x, y \right)=\frac{(2\mu_{x}\mu_{y}+C_{1})({2\sigma}_{xy}+C_{2})}{({\mu^{2}}_{x}{{+\mu}^{2}}_{y}+C_{1})({\sigma^{2}}_{x}+{\sigma^{2}}_{y}+C_{2})}$$

Here, $\mu_{x}$, $\mu_{y}$, and $\sigma_{x}$, $\sigma_{y}$are the local means and standard deviations for images $x$ and $y$respectively, and $\sigma_{xy}$ is the cross-covariance term for images $x$ and $y.$ $C_{1}$ and $C_{2}$ are stability constants to avoid errors when the delta between images is very small.

# Introducing BASSA

BASSA, an acronym for “Bio-Acoustics Superlet Spectrogram Analyser”, is a desktop software application for time frequency-analysis (TF) and visualisation of complex, low-frequency animal sounds. BASSA implements the Superlet transform (SLT), a newly introduced TF analysis algorithm that has been shown to visualise electroencephalograph signals with improved accuracy compared with conventional methods (Moca et al., 2021). Evidence for the SLT’s utility in visualising animal sounds is given in the accompanying paper. To our knowledge, there is currently no GUI-based software tool that employs the SLT for time frequency analysis and visualisation, and therefore the use of the SLT is restricted to those with a certain amount of computer programming skill. BASSA has been therefore designed to make this new algorithm available to researchers in bioacoustics and acoustic ecology, and to improve access to a more accurate method for time frequency analysis of animal sounds.

BASSA’s advantage over existing software lies in its ability to visualise the complex temporal details of features like growls, glottal pulses, and amplitude modulations, while also retaining high resolution spectral details of vocalisations that feature closely spaced tonal components, biphonation, complex frequency modulations, so-called “deterministic chaos”, and nonlinear components. This makes BASSA well suited to the study of vocal production methods, vocal anatomy, and vocal biomechanics. The SLT’s ability to simultaneously resolve high resolution temporal and spectral details also makes BASSA useful in distinguishing subtle differences between similar vocalisations, for the purpose of individual, population, or species identification in acoustic ecology applications.

One of the SLT’s greatest strengths is that it requires minimal tuning to produce optimal visualisations for any arbitrary signal. Producing Fourier-based spectrograms requires careful tuning of the window size, type, and overlap to produce visualisations appropriate for the signal. Additionally, optimal STFT parameters are somewhat dependent on the goals of the analysis, due to the time-frequency resolution trade off discussed in the accompanying paper.

In contrast, the BASSA’s SLT parameters can be left at default values for a wide range of low frequency animal sounds, and tuning is rarely required to retain TF details of interest. BASSA has been designed with this in mind, and while experimentation with the tuning of the SLT algorithm is possible and may be beneficial, highly detailed, accurate visualisations are possible for a wide range of sounds with default settings.

For a detailed explanation of the SLT, please see the Supplementary Material provided by Moca et al. (2021), [found here](https://static-content.springer.com/esm/art%3A10.1038%2Fs41467-020-20539-9/MediaObjects/41467_2020_20539_MOESM1_ESM.pdf). BASSA’s code base is released open-source under the MIT licence, and is available at the project Github [ANONYMISED].

## Software Interface

### The Pre-Processing Tab

BASSA’s graphic user interface is divided into two tabs; the “Pre-Processing” tab, and the “Superlet Scalogram” tab, selected using the tab buttons (Figure 3, items 1 & 2). The pre-processing tab facilitates the loading (Figure 3, item 5) and preparation of audio files for analysis. After loading a file, the time domain waveform of the audio is displayed in the top plotting area (Figure 3, item 3) and the frequency domain magnitude spectrum is displayed in the bottom plotting area (Figure 3, item 4). These displays are intended to provide a guide as to what time and frequency regions contain information of interest.

Pre-processing functions include the ability to trim a recording to the time-region of interest (Figure 3, items 6, 7 & 8) and to resample the audio to a lower sampling frequency, reducing the frequency bandwidth to analyse (Figure 3, items 9, 10 & 11). Trimming and sample rate reduction allow the user to discard times and frequencies that contain no information of interest. This is desirable, as it reduces the amount of data to be analysed, speeding up the computation times, and reduce memory usage. As the signal is trimmed and resampled, the plotting displays update in real-time. Resampling is via a multistage finite impulse response sample rate converter. Output rate tolerance is fixed at 0.01% of the specified sample rate. Zero padding is applied to the end of the signal if the length of the signal is not divisible by the decimation rate.

The pre-processing window also includes the ability to normalize the signal (Figure 3, Item 12), which rescales the amplitude of the time domain signal such that the maximum, absolute value is equal to 1. In cases where absolute sound pressure levels are not required, or pressure sensitivity calibration data for recording equipment is not available, normalization of this type is common practice. Other functionality on this tab includes axis and unit scaling options for the magnitude spectrum plot (Figure 3, Items 13 & 14), and the ability to export the plots in a variety of image file formats (Figure 3, Items 16 & 17). Figure 3, item 15 resets the audio signal to its original duration, sample rate and amplitude.

### The Audio Playback Panel

The playback panel at the bottom of the window is present for both the pre-processing and Superlet scalogram tabs, and allows control over the volume (Figure 3, Item 19), the playback speed (Figure 3, Item 20), single play or continuous looped playback (Figure 3, Item 21) and selection of the audio output hardware device (Figure 3, Item 22). Figure 3 Item 18 starts and stops playback, and other controls are disabled while the audio is playing. The signal referenced and modified by the audio playback panel is an independent copy of the loaded signal. If trimming has been applied in the pre-processing tab, this is reflected in the playback copy, but resampling is not. Changes made to volume or playback speed in the playback panel are not propagated to SLT analysis, or to the plot displays in the pre-processing tab.


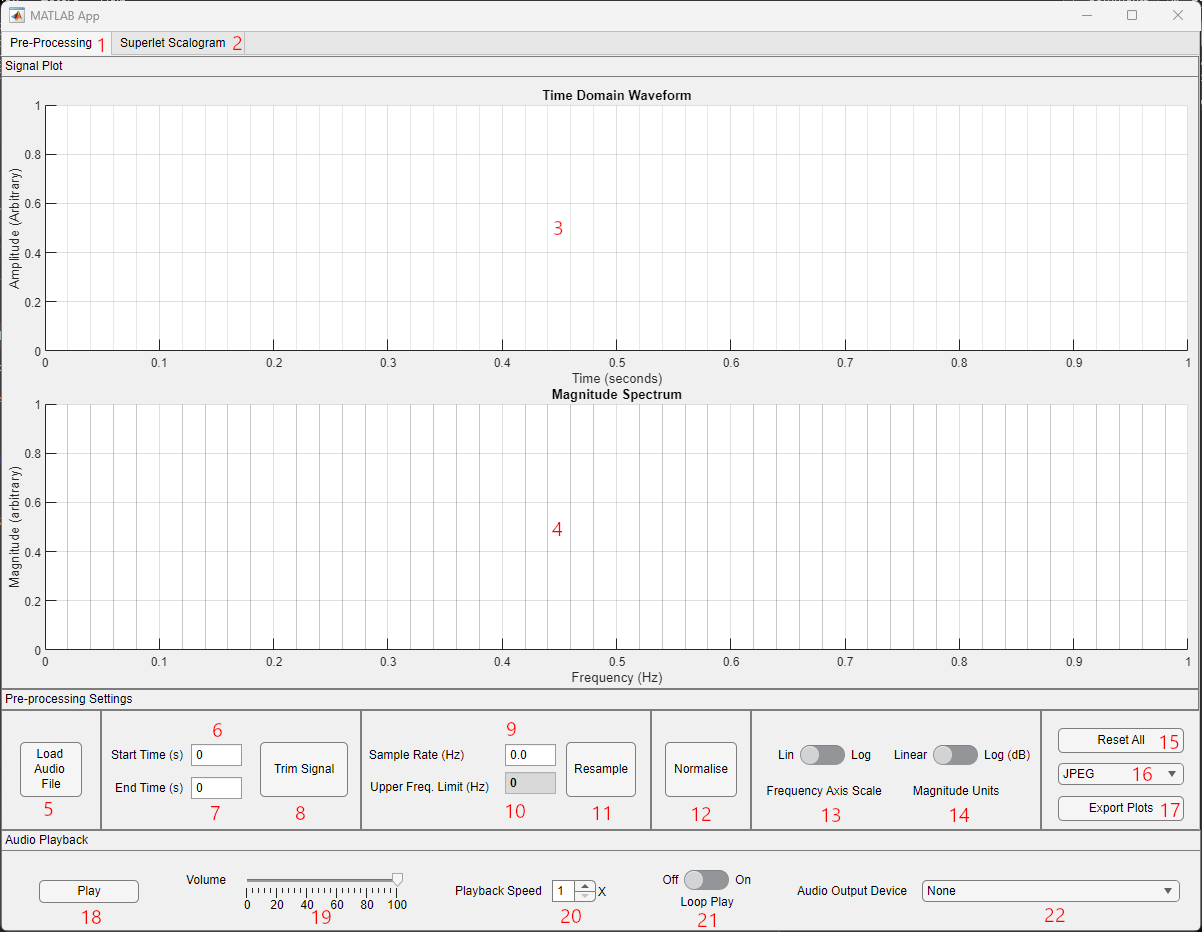


Figure 3 - The Pre-Processing Tab of BASSA. Elements of the interface are numbered in red and referenced throughout the text.

### The Superlet Scalogram Tab

The Superlet Scalogram Tab features a scalogram plotting display (Figure 4, Item 1), SLT algorithm parameters (Figure 4, Items 2-4 & 8-11), signal intensity unit conversions (Figure 4, Items 13-16), and plotting controls (Figure 4, Items 19-25), including a “dynamic range” control (Figure 4, Item 19) that has the effect of de-emphasising low-intensity sounds in the visualisation, effectively increasing the signal-to-noise ratio and making the loudest signals easier to distinguish from background noise. The computation and plotting of the SLT is triggered by the “plot scalogram” button (Figure 4, Item 12). Note that the scalogram intensity units are currently always normalised such that the maximum value is equal to 1 for linear scaled intensity and 0 for logarithmically scaled intensity (dB).

Three other items appear in the “Algorithm Settings” panel; the “Time Resolution” edit field, and the “Max. Time Resolution” and “Estimated RAM to Render” fields, which are read-only. These three fields do not relate to the SLT algorithm directly, but rather to a data reduction procedure that follows the computation of the SLT. The Superlet transform returns an $m\times n$ matrix of data, where $m$ is the number of frequency points in the analysis, defined by the between the lowest and highest frequencies of interest controls, and the frequency resolution control, and $n$ is the number of samples in the time domain signal. The size of $n$ is the product of signal duration in seconds, and sample rate in Hz. For most signals, this means the result of the SLT is massively redundant in terms of temporal sample points, containing far more information than is required to capture the features of interest. For example, a signal with a sample rate of 500Hz and a duration of 200 seconds will result in an SLT scalogram matrix with 100,000 columns, each of which represents a time step of 0.002 seconds.

Many of the complex temporal features in animal vocalisations have durations that are orders of magnitude longer than this. For example, the pulse rate of Unit 2 of the SEP2 song of the Pacific blue whale (*Balaenoptera musculus*) is approximately 6 Hz, equating to a pulse duration of around 0.166 seconds (Malige et al., 2020), while the pulsed call of the striped possum (*Dactylopsila trivirgata*) has a pulse rate of approximately 10 Hz, and a pulse duration of around 0.1 seconds (Volodin & Volodina, 2002). Unlike a time-domain signal, reducing the number of sample points on the time-axis of a signal in the time-frequency domain does not result in frequency aliasing, but simply reduces the density of time instants in the visualisation. Assuming the selected time resolution value is shorter than the duration of the shortest temporal feature of interest, the loss of those samples is inconsequential. Therefore, in the interest of reducing memory utilisation and compute times, the data reduction procedure takes the raw SLT output and performs a nearest neighbour interpolation, reducing the number of columns of the SLT scalogram matrix according to the value set by the time resolution control (Figure 4, item 5). The default value of this control (in seconds) is set dynamically according to the following:

$${tres}_{default}=\frac{d}{2\cdot fs}$$

Where $d$ is the total duration of the signal being analysed (in seconds), and $fs$ is the sampling frequency in Hz (after resampling on the pre-processing tab, if applicable). The user can increase or decrease time resolution, with the maximum possible value being shown in the read-only field (Figure 4, Item 6). The total size of the SLT scalogram matrix is determined by the time and frequency resolution settings, settings for the lowest and highest frequencies of interest, and by the duration of the signal. A live updating, read-only field (Figure 4, Item 7) gives an estimate of the memory required to compute this matrix and pass it to the graphics renderer.

Converting linear scaled magnitude or power units to decibels involves taking the logarithm of intensity, and for intensity values that approach the smallest computable floating-point number, taking the logarithm may result in “-inf” values. To avoid this, the function that performs unit conversions in the SLT tab searches the SLT scalogram matrix for the smallest non-“inf” value after taking the logarithm. It then replaces all values of “-inf” in the matrix with this minimum value. There is therefore some information lost at the very lowest intensities, however in most test cases this was shown to occur at 70 – 90 dB below the signal of interest, and so the information lost is assumed to meaningless background noise.


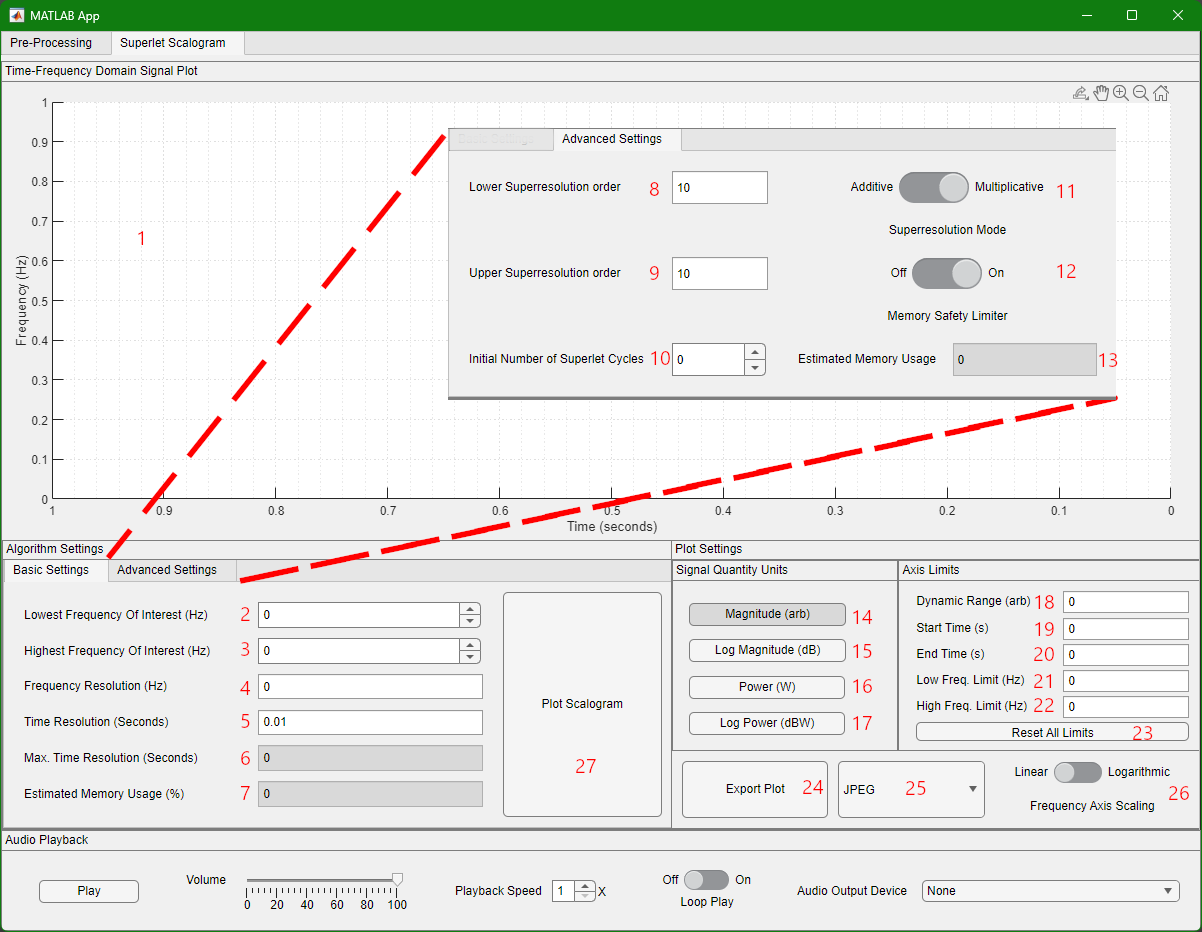


Figure 4 - The Superlet Scalogram Tab of BASSA. Elements of the interface are numbered in red and referenced throughout the text.

## Limitations

BASSA was developed in the MATLAB™ App Designer, which places certain limitations on memory usage within the application. The Superlet Transform is a computationally complex algorithm, and while several of its parameters influence compute times and memory usage, the primary determinant of computational expense is the sampling frequency of the input signal, the second being the signal’s duration. As such, the present implementation of BASSA has limited ability to analyse long duration, high sample rate signals. Currently the maximum sample rate for signal analysis is 1kHz, and the maximum duration is 80,000 samples. Audio files exceeding these limits can be loaded into BASSA’s pre-processing page, then trimmed and re-sampled before progressing to the Superlet Scalogram Tab.

Even within these limits, users are encouraged to use workflows that maximise memory efficiency, such as trimming irrelevant time regions from the signal and reducing the sample rate such that the signal contains only information within the frequency range of interest. Using temporal and spectral resolutions only as high as necessary to resolve the salient features of the signal is also recommended to reduce computational load. The BASSA user guide (available at the [ANONYMISED] repository) gives step by step instructions for such a workflow. A memory usage estimate has been built into the software to prevent users from exceeding the available memory, and warnings will occur if the signal has excessively long duration or high sample rate. The memory limiter can be overridden in the advanced panel of the SLT parameters, but this will likely result in unexpected behaviour including extremely slow rendering of figures, hangs and complete crashes.

BASSA currently only supports single channel audio analysis. If a stereo or multichannel file is loaded, a dialog box prompts the user to select the channel number to be analysed. Other channels are discarded.

The intensity values of the SLT visualisations are normalised to the maximum value, this is necessary due to complexity in the order of operations for unit conversions, and to avoid unexpected behaviour when taking the logarithm of very small values. Currently BASSA does not feature the ability to calibrate intensity values to the pressure sensitivity of the recording equipment, so absolute sound pressure levels cannot be plotted. This may be added in a future release.

The following audio file formats are supported: AIFC, AIFF, AU, FLAC, OGG, OPUS, WAVE, MP3, MPEG-4, and AAC. Plots can be exported in the following image file formats: JPEG, PNG, TIFF, PDF, and EPS.

## Performance Benchmarks

BASSA has been tested with a series of audio files varying durations and sample rates. Table 1 gives benchmarking times the computation and rendering of SLT’s for these audio files. These tests were performed on a 2023 model Dell Precision 3570 laptop running Windows 11, with 64GB of memory, an Intel i7-1265U CPU and an Nvidia T550 Laptop GPU.

Table 1: Compute and render time benchmarks. “Freq. Res.” refers to frequency resolution. “Time. Res.” refers to time reolution. FOI refers to frequency of interest. All other SLT parameters not listed in this table were set at default values.

| Duration (s) | Sample Rate (Hz) | Time Res. (s) | Freq. Res. (Hz) | Lowest FOI (Hz) | Highest FOI (Hz) | Compute + Render Time (mm : ss) |
| --- | --- | --- | --- | --- | --- | --- |
| 60 | 1000 | 0.05 | 0.5 | 10 | 499 | 06:27 |
| 120 | 500 | 0.01 | 0.25 | 10 | 249 | 01:21 |
| 240 | 250 | 0.005 | 0.25 | 10 | 124 | 00:46 |

## Future Development

Several improvements are planned for future releases of BASSA. These include:

- Improved performance.
- Pressure sensitivity calibration offset to allow results in absolute SPL.
- Optional normalisation in the SLT result.
- The ability to analyse longer duration.
- The ability to analyse higher sample rate audio.
- Batch file analysis with macros.

The development of BASSA is a volunteer effort, and the software is released open-source under the MIT licence.

# References

Malige, F., Patris, J., Buchan, S. J., Stafford, K. M., Shabangu, F., Findlay, K., Hucke-Gaete, R., Neira, S., Clark, C. W., & Glotin, H. (2020). Inter-annual decrease in pulse rate and peak frequency of Southeast Pacific blue whale song types. *Scientific Reports*, *10*(1), 8121. https://doi.org/10.1038/s41598-020-64613-0

Moca, V. V., Bârzan, H., Nagy-Dăbâcan, A., & Mureșan, R. C. (2021). Time-frequency super-resolution with superlets. *Nature Communications*, *12*(1), Article 1. https://doi.org/10.1038/s41467-020-20539-9

Volodin, I. A., & Volodina, E. V. (2002). Uninterrupted vocalization in the striped possum Dactylopsila trivirgata (Marsupialia, Petauridae) during the whole respiratory cycle. *Zoologicheskii Zhurnal*, *81*(12), 1526–1529. Scopus.

# BASSA User Guide


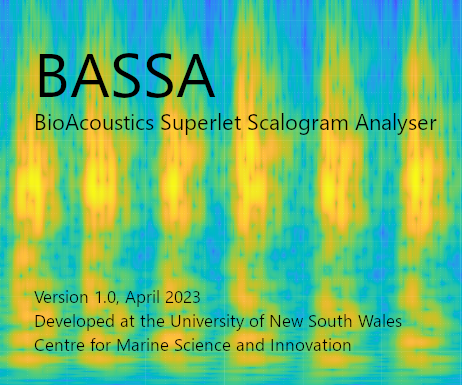


Table of Contents

[Introduction 2](#_Toc128656636)

[1. System Requirements & Installation 3](#_Toc128656637)

[2. Supported Audio Import File Formats 3](#_Toc128656638)

[3. Supported Image Export File Formats 3](#_Toc128656639)

[4. The User Interface 4](#_Toc128656640)

[4.1. The Tab Bar 4](#_Toc128656641)

[4.2. The Pre-Processing Tab 4](#_Toc128656642)

[4.3. The Audio Playback Panel 5](#_Toc128656643)

[4.4. The Superlet Scalogram Tab 6](#_Toc128656644)

[5. Further Information 8](#_Toc128656645)

[5.1. The Audio Playback Panel 8](#_Toc128656646)

[5.2. Interactive Plots 8](#_Toc128656647)

[5.3. A Note on Performance and Memory Usage 8](#_Toc128656648)

[5.4. Superlet Parameters: Some Technical Details 9](#_Toc128656649)

# Introduction

BASSA is a tool for time-frequency analysis of audio signals. It was designed to facilitate the visualisation of low frequency animal sounds with greater accuracy and detail than conventional short-time Fourier spectrogram methods. Fourier-based time-frequency visualisations are known as spectrograms, whereas BASSA produces “Scalograms”. Scalograms look like spectrograms but are computed using wavelet-based algorithms. The specific algorithm underlying the scalograms produced by BASSA is the “Superlet transform” also known as the SLT (Moca et al., 2021).

The SLT is appropriate for time-frequency analysis of any low frequency animal sound, though it excels most when applied to complex sounds, and is particularly adept at visualising the fine temporal details in glottal pulses, amplitude modulations, noisy, and non-linear sounds, while also retaining high resolution frequency details. This makes BASSA especially useful in the study of vocal production methods, vocal anatomy and vocal biomechanics. While the suggested applications lie in bioacoustics, BASSA is useful for time-frequency analysis of any time-series data, from human speech, to brainwaves, to seismographic data.

One of the SLT’s greatest strengths is that it requires minimal tuning to produce optimal visualisations for any arbitrary signal. Fourier-based spectrograms require careful tuning of their window size, type, and overlap to produce useful visualisations. Those parameters must be optimised for each signal of interest, and depend on the goals of the analysis, which might be, for example, to prioritise temporal details of a sound. This is not the case for the SLT, which can produce accurate visualisations for a wide range of signals, with no parametric tuning. The user interface of BASSA has been designed with this in mind, and while tuning of the SLT is possible, dynamic optimisation of default algorithm values means manual tuning is not strictly necessary. For deeper explanation of SLT parameters, see 5.4. Superlet Parameters: and the original “Superlets” publication: [Time-frequency super-resolution with superlets](https://www.nature.com/articles/s41467-020-20539-9).

BASSA was designed at the MammalLab, in the Evolution and Ecology Research Center, University of New South Wales, Sydney, Australia.

## System Requirements & Installation

- Operating System: Windows 11 (64-bit)
- Processor: Intel or AMD x64 processor with four logical cores
- Memory (RAM): 16 GB or more
- Storage: At least 2 GB of free disk space
- Display: 1366 x 768 or higher resolution monitor

Earlier versions of Windows may be compatible but are currently untested. Some functionality may be inoperable or unstable on earlier operating systems. Virtual machines also have not been tested.

Other requirements:

- MATLAB Compiler Runtime (MCR) version 9.9. The BASSA installer will download and install this automatically if not already installed.
- Microsoft .NET Framework 4.8 or later.

It is recommended to have a stable internet connection to download and install the application, as well as any necessary updates. Please note that some anti-virus or firewall software may interfere with the installation or functioning of the MCR.

## Supported Audio Import File Formats

Note that BASSA currently supports only single channel audio. If a stereo or multi-channel audio file is loaded, a dialog box gives the user the option to select which channel to work on.

- AIFC (.aifc)
- AIFF (.aiff, .aif)
- AU (.au)
- FLAC (.flac)
- OGG (.ogg)
- OPUS (.opus)
- WAVE (.wav)
- MP3 (.mp3)
- MPEG-4 AAC (.m4a, .mp4)

## Supported Image Export File Formats

- JPEG (.jpg)
- PNG (.png)
- TIFF (.tif)
- PDF (.pdf)
- EPS (.eps)

# The User Interface


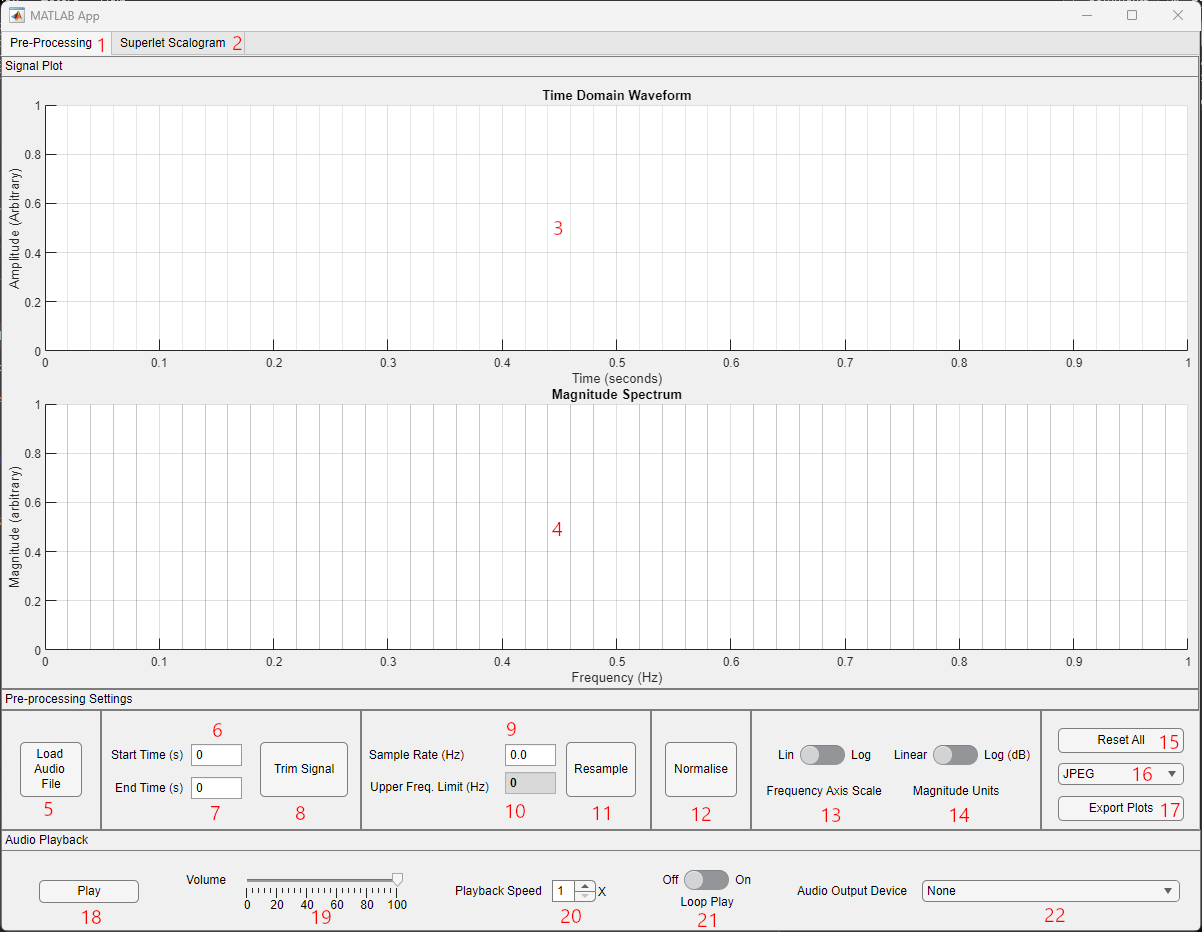


## The Tab Bar

1. Pre-processing tab select button.
2. Superlet scalogram tab select button.

## The Pre-Processing Tab

1. Waveform plot area. This shows the signal that will be analysed by the SLT in the time domain.
2. Magnitude spectrum plot area. This shows the signal that will be analysed by the SLT in the frequency domain.
3. Load Audio File Button. Opens a file load dialog box.
4. Edit audio start time (in seconds). Disabled after trimming, re-enabled by “Reset All”.
5. Edit audio end time (in seconds). Disabled after trimming, re-enabled by “Reset All”.
6. Trim audio signal to be analysed by the SLT, according to the new start and end times. Changes made to the signal start and end times are reflected in the signal sent to the audio playback panel. Disabled after trimming, re-enabled by “Reset All”.
7. Edit the audio signal’s sample rate. The lower the sample rate of the audio, the faster the SLT analysis will be. Sample rate determines the highest frequency that can be analysed. Disabled after resampling, re-enabled by “Reset All”.
8. Read-only: The new highest frequency that will be analysed as per the new sampling rate.
9. Resample the audio signal to be analysed by the SLT, according to the new sample rate. Changes to the sample rate do not affect the audio signal sent to the playback panel. Disabled after resampling, re-enabled by “Reset All”.
10. Normalise the audio signal to be analysed by the SLT, and the magnitude spectrum plot, so that the maximum amplitude and magnitude values are equal to 1. This is recommended when absolute source amplitude information is not required, or recording equipment sensitivity calibration values are not known. This does not affect the audio signal sent to the playback panel. Disabled after normalising, re-enabled by “Reset All”.
11. Re-scale the magnitude spectrum plot so that the frequency axis (y-axis) is logarithmically spaced. This has no effect on the SLT.
12. Switch the scaling of the magnitude spectrum y-axis units from linear magnitude to logarithmic magnitude (aka. Decibels/dB). This has no effect on the SLT.
13. Reset the audio signal start and end times, sample rate and amplitude values to their original values. This also clears any data in the SLT tab and returns settings there to default values.
14. Select the image file format for plot exports.
15. Export the time domain waveform plot and frequency domain magnitude spectrum plots as images.

## The Audio Playback Panel

1. Play the audio signal. One click (button down) starts playback, click again (button up) to stop playback.
2. Set the playback volume. Changes to this setting do not affect the signal to be analysed by the SLT.
3. Set the playback speed. Increasing playback speed is common practice to make very low frequency signals easier to hear on conventional speakers. Changes to this setting do not affect the signal to be analysed by the SLT.
4. When this switch is set to “on”, playback of the signal will run in a continuous loop until the play button or the loop switch are set to “off”.
5. Select the audio output hardware device. Default will be the current Windows playback device.


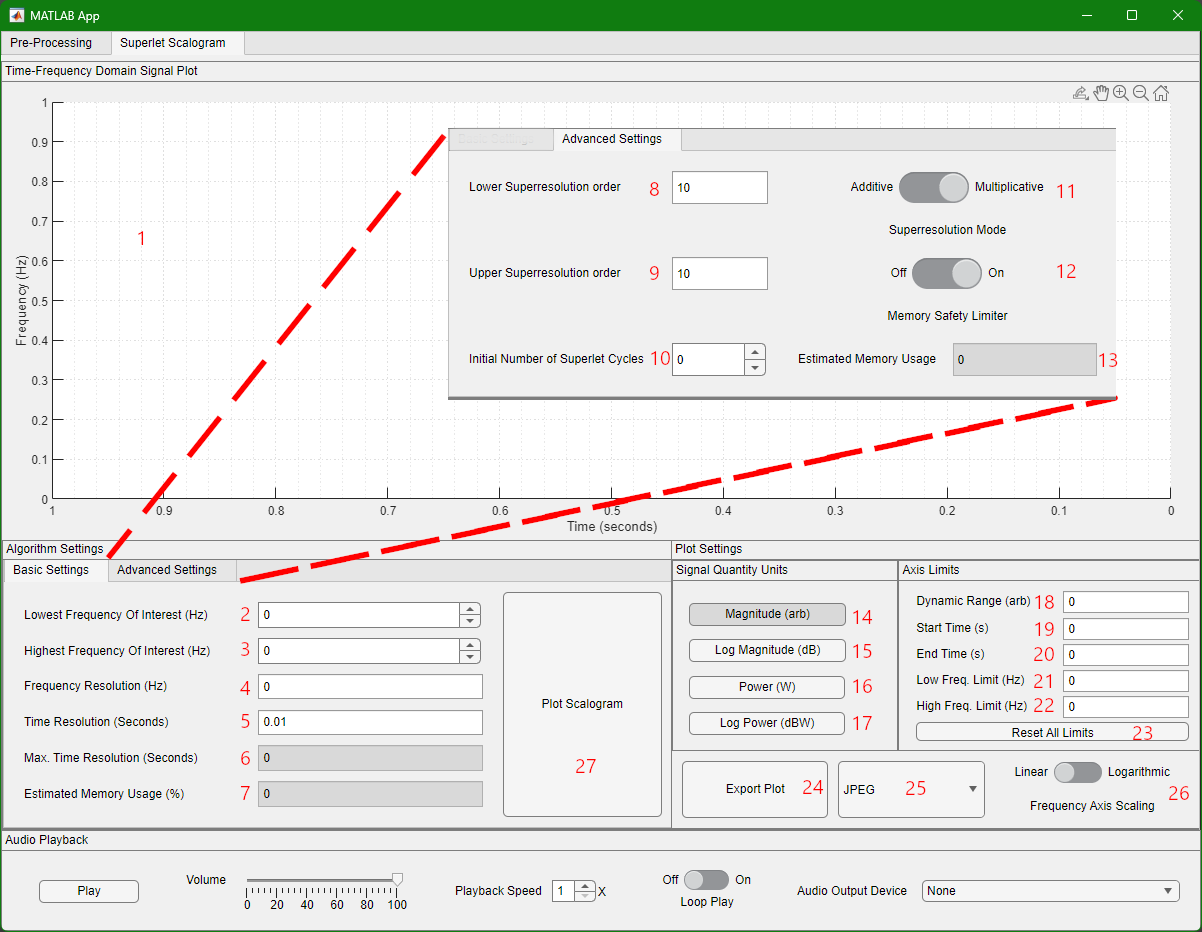


## The Superlet Scalogram Tab

1. Superlet Scalogram plot area. The results of the time-frequency analysis are visualised here.
2. The lowest frequency analysed by the SLT. Default is 10 Hz.
3. The highest frequency analysed by the SLT. Default is half of the sample rate.
4. The size of the frequency steps in the SLT analysis. A smaller number indicates higher resolution. Default is 0.25 Hz.
5. The size of the time steps in the SLT analysis. A smaller number indicates higher resolution. Default is the signal duration in seconds, divided by two times the sample rate.
6. The maximum possible time resolution (determined by the sample rate of the audio).
7. An estimate of the memory used to compute and render the visualisation.
8. The lower limit of superresolution orders. Recommend using default setting of “10”. (See below for further explanation)
9. The upper limit of superresolution orders. Recommend using default setting of “40”. (See below for further explanation)
10. Number of cycles in the base wavelet of a Superlet set. Recommend using default setting of “4”. (See below for further explanation)
11. Switch between additive or multiplicative superresolution. Recommend using default setting of “multiplicative”.
12. Memory Limiter Override Switch. Not recommended. May cause BASSA to crash or freeze.
13. Same as (7)
14. Set plot intensity units to unitless magnitude, normalized such that maximum dynamic range is 0 to 1.
15. Set plot intensity units to the logarithm of magnitude (Decibels/dB), normalized such that maximum dynamic range is -inf to 0.
16. Set plot intensity units to unitless Power, normalized such that maximum dynamic range is 0 to 1.
17. Set plot intensity units to the logarithm of Power (20*log10(magnitude)), normalized such that maximum dynamic range is -inf to 0.
18. Reduce the dynamic range of the SLT plot. This increases the lower axis limit of the colour bar, which represents intensity. It can help to de-emphasise low intensity sounds like background noise and can improve the visualisation of the signal of interest. Units follow the selections made in the “Signal Quantity Units” panel. E.g., reducing this value to 60dB with units set to mag dB will plot all intensities smaller than the maximum value minus 60dB, using the colour of the minimum value in the figure’s colour axis.
19. Edit the time axis lower limit. This affects the plot only, and does not recalculate the SLT. The complete scalogram data remains intact when axis limits are reduced.
20. Edit the time axis upper limit. This affects the plot only, and does not recalculate the SLT. The complete scalogram data remains intact when axis limits are reduced.
21. Edit the frequency axis lower limit. This affects the plot only, and does not recalculate the SLT. The complete scalogram data remains intact when axis limits are reduced.
22. Edit the frequency axis upper limit. This affects the plot only, and does not recalculate the SLT. The complete scalogram data remains intact when axis limits are reduced.
23. Reset all plot axis limits.
24. Export the SLT Scalogram plot as an image.
25. Select the file type of the exported plot.
26. Re-scale the SLT plot so that the frequency axis (y-axis) is logarithmically spaced. This can improve visibility of closely spaced low frequency components.
27. Click to plot the scalogram using the current settings. Can be clicked again to re-plot with new settings.

# Further Information

## The Audio Playback Panel

Any audio file that is loaded with a sampling rate that is higher or lower than the rates supported by the selected sound card are re-sampled to the closest supported sampling rate. This resampled version of the signal is used for audio playback only, and is not seen by the Superlet Scalogram tab.

The playback speed changes the sample rate of the audio output device. The file is not modified. The playback audio is always normalised such that the peak absolute amplitude is equal to 1. Volume adjustments are applied to normalised audio. This avoids the possibility of clipping the audio output.

## Interactive Plots

All plots in BASSA have interactive functionality. The responsiveness of these functions is highly dependent on memory usage.

**Datatips:** Clicking on a plotted line or surface sets a datatip, which will display values at those plot axis coordinates. For the waveform plot, X = time, Y = amplitude. For the magnitude spectrum plot, X = frequency, Y = magnitude. For the SLT plot, X = time, Y = frequency, Z = intensity. Right clicking a datatip opens a context menu with options for deleting current or all datatips.


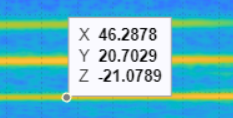


**Interactive Plot Controls:** Hovering the mouse over the plot area will show several tool icons in the top right-hand side of the plot. These tools are, from left to right: Save/Export, rotate in 3D, Pan, Zoom in, Zoom out & Return to original view.


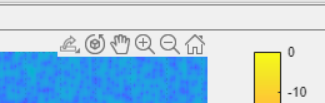


## A Note on Performance and Memory Usage

BASSA was designed in the MATLAB App Designer, which uses the Chromium Embedded Framework (CEF) to render graphics. The CEF has a maximum JavaScript heap size of a little less than 4GB, and there is currently no way to increase the amount of memory it can use. This places an unfortunate limitation on BASSA’s ability to plot very large SLT scalograms. Future MATLAB updates may resolve this, however at present, it is recommended that analysis settings be optimised for memory efficiency. Memory-saving default values have been set for the relevant controls. These can be overridden, but you may experience performance issues or instability.

Long audio files, files with a high sample rate, or analysing files across a large frequency range, or with very high time or frequency resolution will all cause memory use to skyrocket, and visualisations may take a very long time to compute and visualise. In extreme cases, this may cause BASSA to become unresponsive or crash entirely.

In testing, BASSA has been shown to operate normally with an audio file of 200 seconds in duration, with a sample rate of 500Hz, no trimming, no resampling, and all default SLT settings. The SLT in this test case took approximately 2 minutes to compute and plot on machine running Windows 11, with 64GB system memory, an Intel i7-1265U and an Nvidia T550 Laptop GPU.

To minimise memory usage issues, the following workflow is recommended:

1. Load an audio file.
2. Using the waveform plot as a guide, edit the start and end times and trim the audio, discarding region that contain no signals of interest.
3. Using the magnitude spectrum plot as a guide, determine the highest frequency that contains information of interest. Switching magnitude units to dB may be helpful here.
4. Reduce the sample rate to a value that is 2x the highest frequency of interest. E.g., if the highest information of interest in the audio signal occurs at 90 Hz, resample to 200 Hz, which will result in an upper frequency limit of 100 Hz.
5. Normalise the signal. Not a memory saver, just good practice.
6. Switch to the Superlet Scalogram Tab.
7. Inspect the memory estimate field.
8. If the memory estimate is above 3000 MB, it is recommended to reduce time or frequency resolution. While the default value for time resolution is calculated dynamically to optimise for memory efficiency, for many signals it may still end up being higher than necessary. Time resolution can often be reduced by an order of magnitude without losing meaningful temporal detail. For many animal sounds, a time resolution of 0.1 seconds is more than enough. Frequency resolution too, can often be reduced.
9. Use the edit fields for highest and lowest frequency of interest to further reduce the number of frequencies to be computed.
10. Recheck the memory estimate field. It will update when changes are made to frequency range and resolution settings.
11. Click “plot scalogram”.

## Superlet Parameters: Some Technical Details

The Superlet transform is a generalisation of the continuous wavelet transform (CWT). The CWT determines the amount of energy at the frequency of interest is present in a signal, by convolving it with a “wavelet” whose centre frequency is at the frequency of interest. The initial “mother” wavelet has a specified number of cycles, and for each frequency of interest the mother wavelet is compressed in time, increasing its centre frequency while the number of cycles is held constant. The time resolution of the CWT is determined, in part, by the number of cycles in the mother wavelet.

The SLT operates by computing a plurality of continuous wavelet transforms, each having a different number of cycles in the mother wavelet. It then takes the geometric mean of the resulting CWT scalograms, to produce an SLT Scalogram.

The specific numbers of cycles used in the CWT set is determined by the frequency range, frequency resolution, initial number of Superlet cycles, the superresolution order interval, and whether the SLT is operating in additive or multiplicative mode.

**Frequency Range & Resolution:** The wider the frequency range and the higher the resolution (ie. the smaller and more numerous the frequency steps), the more CWTs are required to analyse the entire signal.

**Initial Number of Superlet Cycles:** The number of cycles in the mother wavelet of the initial CWT.

**Multiplicative or Additive Mode:** Determines whether the number of cycles for each CWT’s mother wavelet is calculated by using superresolution orders as multiplication factors or addition constants.

**Upper and lower superresolution order:** This defines the maximum and minimum number of cycles. If the frequency domain contains 0 Hz, then the lower order refers to frequency 0 and the upper order to the positive (upper) frequency boundary - the order will then mirror around 0 into the negative domain.

# References

Moca, V. V., Bârzan, H., Nagy-Dăbâcan, A., & Mureșan, R. C. (2021). Time-frequency super-resolution with superlets. *Nature Communications*, *12*(1), Article 1. https://doi.org/10.1038/s41467-020-20539-9
